# Supplementary material for: Strong TCRγδ Signaling Prohibits Thymic Development of IL-17A-Secreting γδ T Cells
Source: Cell Rep. 2017 Jun 20;19(12):2469–76. doi: 10.1016/j.celrep.2017.05.071 (PMC5489697; doi:10.1016/j.celrep.2017.05.071)
Supplement: Document S1. Supplemental Experimental Procedures and Figures S1–S4 [file mmc1.pdf]

Cell Reports, Volume *19*

Supplemental Information

Strong TCRγδ Signaling Prohibits Thymic Development of IL-17A-Secreting γδ T Cells

Nital Sumaria, Capucine L. Grandjean, Bruno Silva-Santos, and Daniel J. Pennington

Supplementary Figure 1

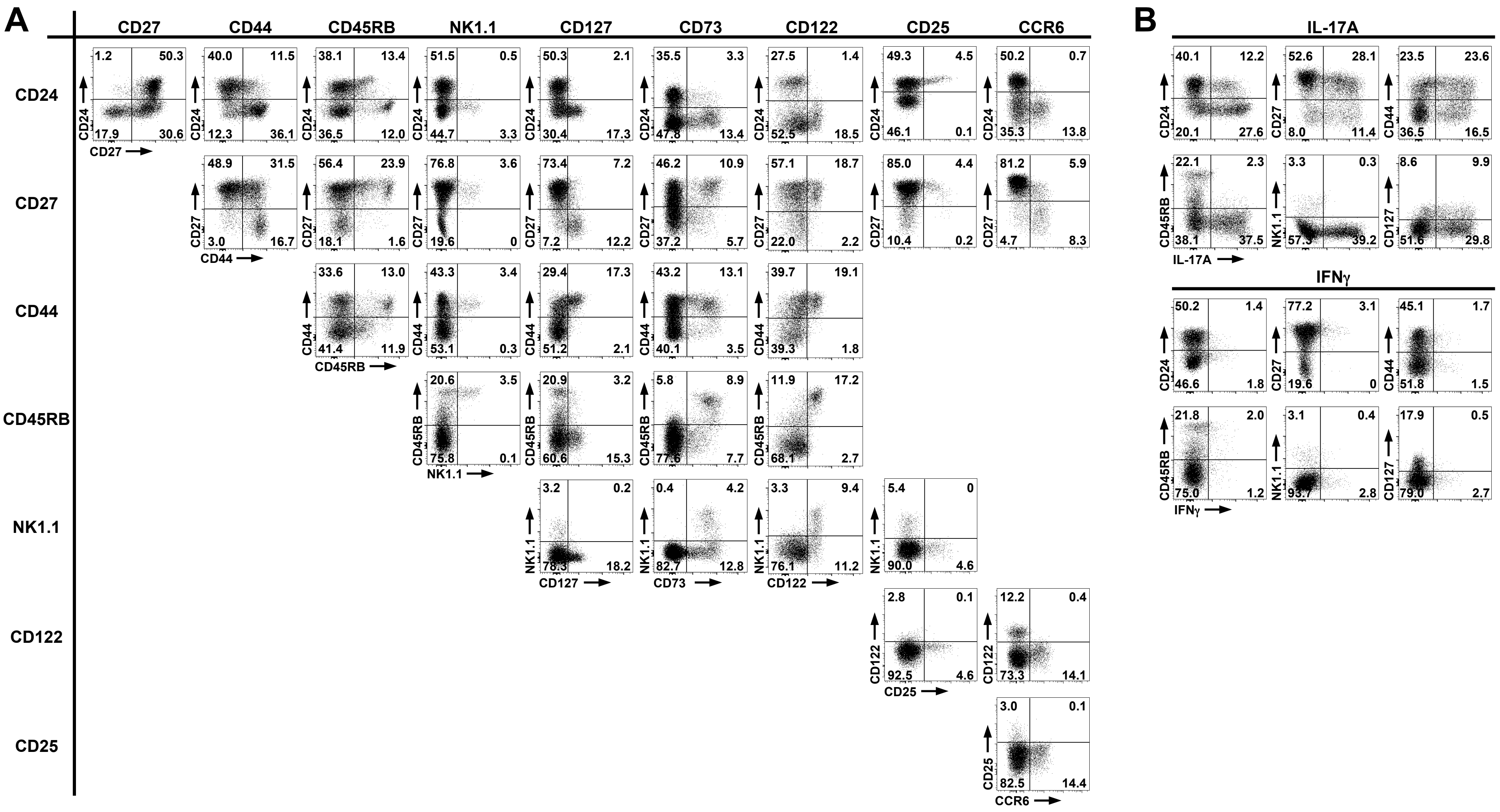

Figure S1. Combinatorial analysis of previously reported surface markers on thymic γδ T cells, Related to Figure 1. (A) Flow cytometry plots of γδ (TCRδ$^{(+)}$CD3ε$^{(+)}$) T cells from the thymus of neonatal C57BL/6 mice ($n \geq 5$), stained for CD24, CD27, CD44, CD45RB, NK1.1, CD127, CD73, CD122, CD25 and CCR6. **(B)** Plots show thymic γδ T cells stained intracellularly for IL-17A and IFNγ in combination with indicated surface markers. Percentages of cells are indicated in each quadrant.

Supplementary Figure 2

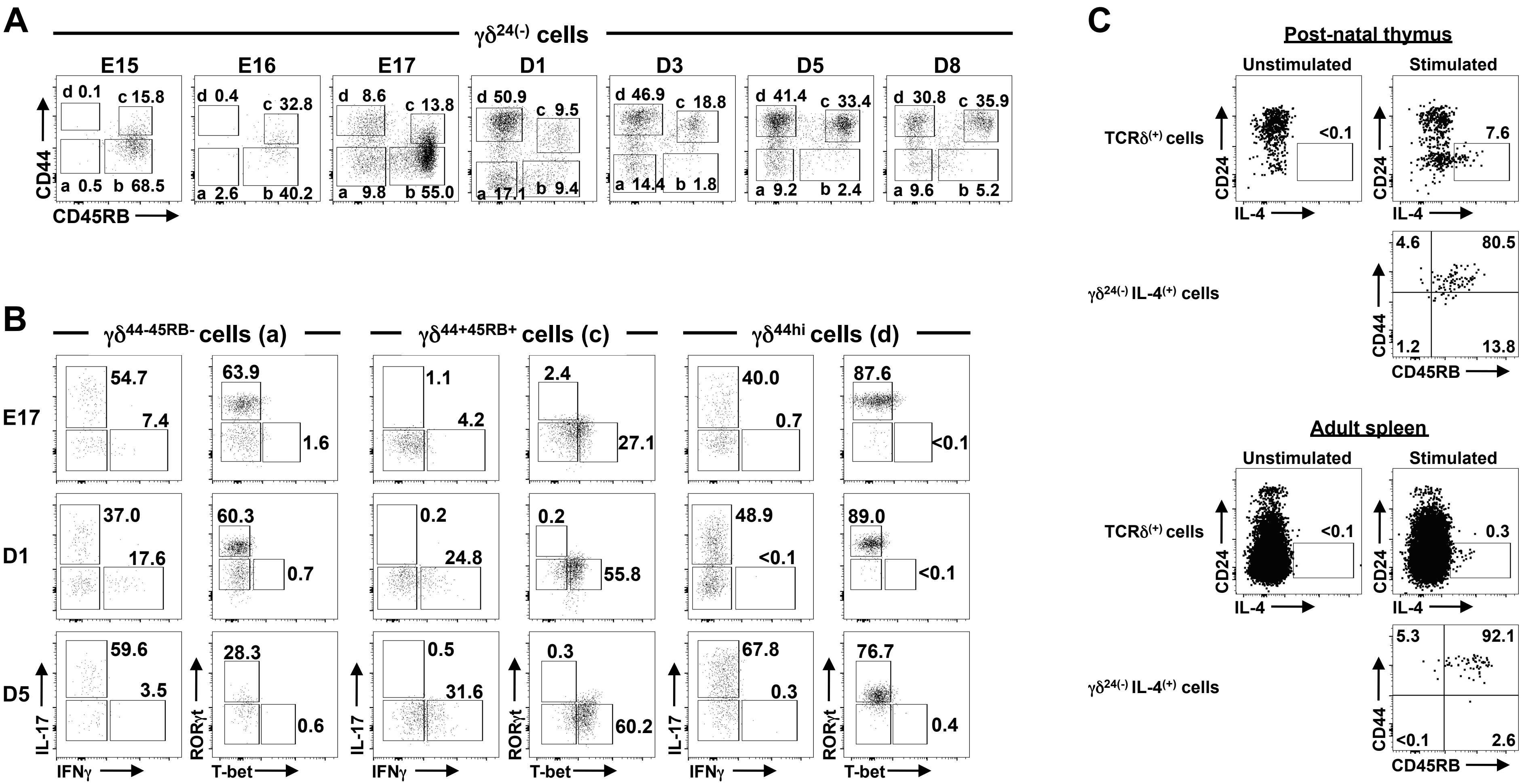

Figure S2. Thymic development of mature γδ T cells and the acquisition of effector function followed through ontogeny, Related to Figure 1. (A) Flow cytometry plots of γδ (TCRδ$^{(+)}$CD3ε$^{(+)}$) T cells in the thymus through ontogeny from E15 to 8-day old B6 mice (*n* ≥ 4 per time-point). CD44/CD45RB plots show CD24$^{(-)}$ γδ T cells. **(B)** Intracellular IL-17A/IFNγ, or RORγt/T-bet, in CD44$^{(-)}$CD45RB$^{(-)}$ (left panel), CD44$^{(+)}$CD45RB$^{(+)}$ (middle panel) and CD44$^{(hi)}$CD45RB$^{(-)}$ (right panel) thymic γδ T cells through ontogeny including E17, 1-day old and 5-day old mice. For cytokines, cells were stimulated 4h *ex vivo* with PMA/ionomycin. **(C)** γδ T cells committed to IL-4 secretion map onto the CD45RB developmental pathway. Total TCRδ$^{(+)}$ thymocytes (top panel) or splenocytes (bottom panel) were sorted from post-natal B6 mice or adult pTα$^{-/-}$ mice, respectively, and stimulated for 18h with PMA/ionomycin or left unstimulated. Top plots in each panel show surface expression of CD24 and intracellular IL-4 staining of γδ T cells. Bottom plots in each panel show surface expression of CD44 and CD45RB on IL-4$^{(+)}$ CD24$^{(-)}$ γδ T cells. Percentages of cells are indicated for each gate or quadrant.

Supplementary Figure 3

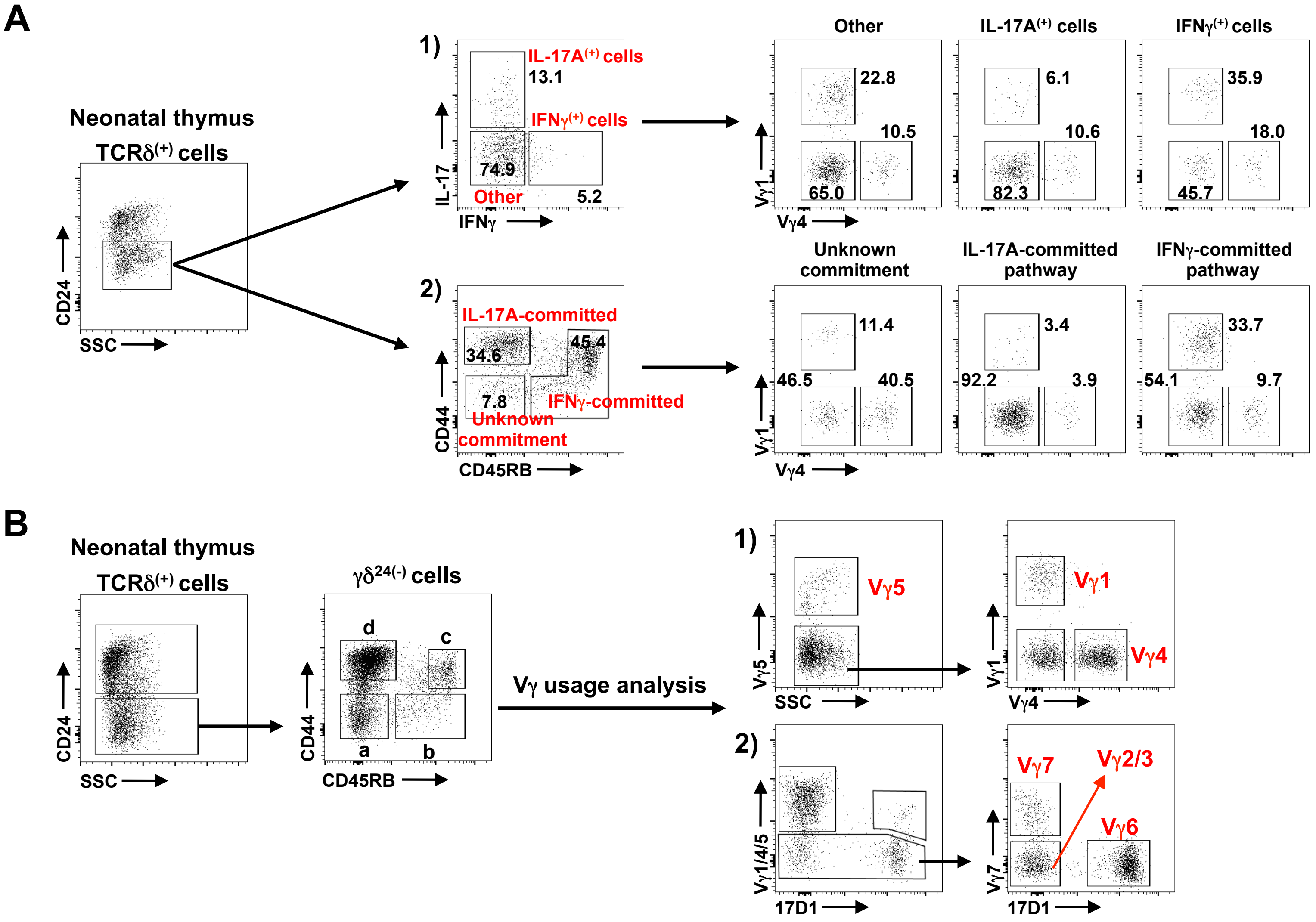

Figure S3. Comparison between gating strategies to identify γδ T cell subsets committed to IL-17A or IFNγ secretion, and specific Vγ usage, Related to Figure 2 and Figure 3. (A) Flow cytometry plots of thymic γδ T cells from neonatal B6 mice stained for CD24, CD44, CD45RB, Vγ1 and Vγ4, followed by intracellular staining for IL-17A and IFNγ. Two gating strategies are depicted: 1) CD24$^{(-)}$ γδ T cells (left) first gated for intracellular IL-17A and IFNγ expression, followed by Vγ-usage (top panels, right) and, 2) CD24$^{(-)}$ γδ T cells (left) first gated for CD44 and CD45RB expression, followed by Vγ-usage (bottom panels, right). Percentages of gated cells are indicated. **(B)** Thymic γδ T cells from neonatal mice were stained using two protocols to identify cells expressing specific Vγ-chains: 1) CD24$^{(-)}$ γδ T cells directly assessed for Vγ1, Vγ4 and Vγ5, and: 2) CD24$^{(-)}$ γδ T cells first gated for Vγ1$^{(-)}$Vγ4$^{(-)}$Vγ5$^{(-)}$ cells followed by analysis for Vγ7 and Vγ6 (using antibody 17D1 that recognises both Vγ5$^{(+)}$ and Vγ6$^{(+)}$ cells).

Supplementary Figure 4

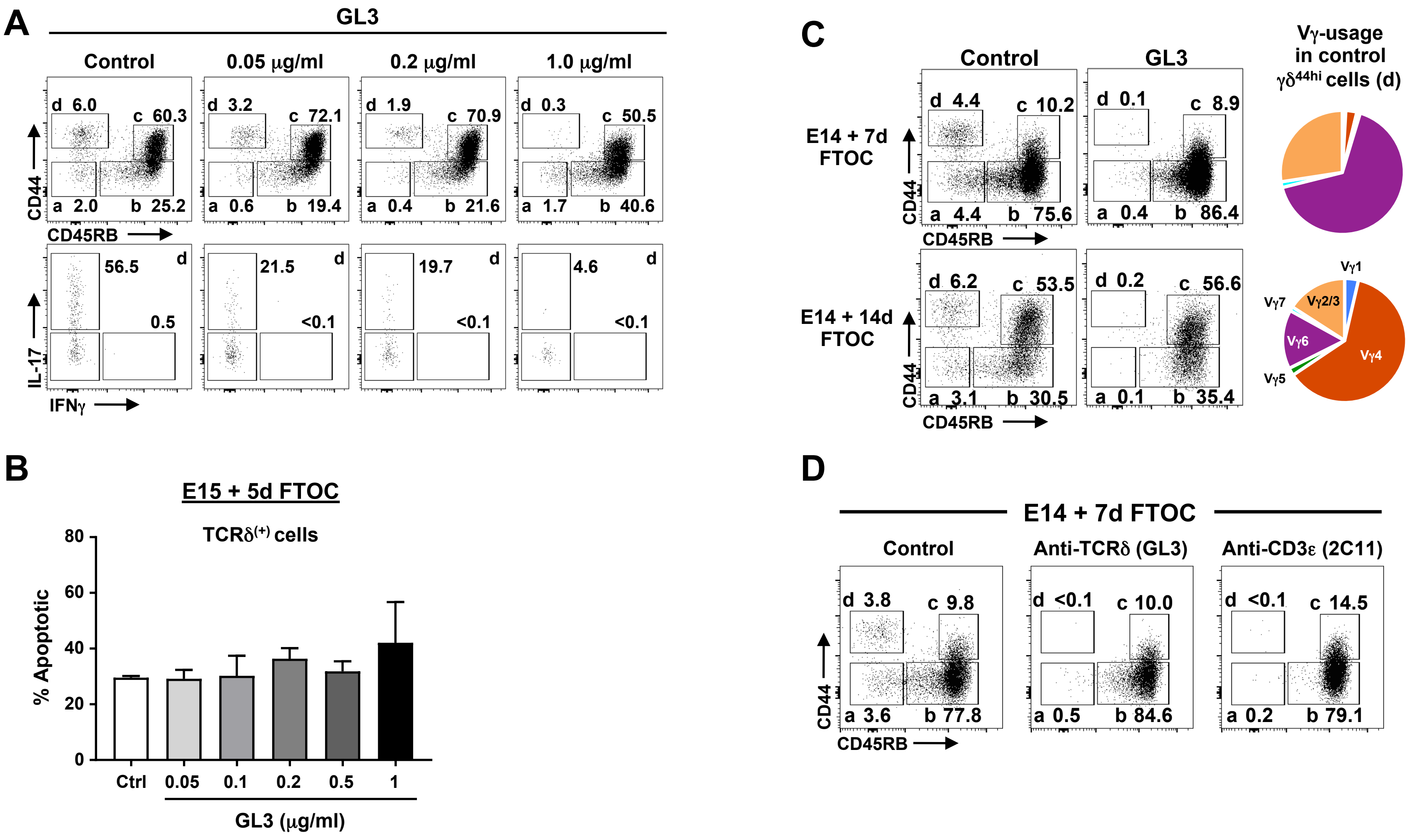

Figure S4. Strong TCRγδ signalling prohibits development of IL-17A-committed γδ T cells, Related to Figure 4. (A) CD44/CD45RB plots (top row) of CD24$^{(-)}$ γδ T cells from 7-day FTOC of E15 B6 thymic lobes in the presence (concentrations as indicated) or absence of anti-TCRδ antibody GL3. Bottom plots show intracellular staining of CD44$^{(hi)}$CD45RB$^{(-)}$ "d" γδ T cells for IL-17A and IFNγ. **(B)** Bar graph shows percentage apoptosis of total γδ T cells from 5-day FTOC of E15 B6 thymic lobes in the presence (concentrations indicated) or absence of GL3. **(C)** CD44/CD45RB (left) on CD24$^{(-)}$ γδ T cells from E14 7-14-day FTOC +/- GL3 (1µg/ml). Vγ-usage by CD44$^{(hi)}$CD45RB$^{(-)}$ γδ T cells is summarised in the pie-charts (right). **(D)** CD44/CD45RB on CD24$^{(-)}$ γδ T cells from 7-day FTOC of E14 B6 thymic lobes in the presence of anti-TCRδ antibody GL3 (1µg/ml), anti-CD3ε antibody 2C11 (1µg/ml), or under control conditions. Percentages of gated cells are indicated. Summarized data are represented as mean ± s.d.

SUPPLEMENTAL EXPERIMENTAL PROCEDURES

Mice: C57BL/6 (B6) mice were purchased from Charles River Laboratories. pTα mutant mice (pTα$^{-/-}$) on a C57BL/6 background were bred in-house. All mice were foetal (E14-E17), neonatal (1-3 days), post-natal (4-8 days) or adult (8-12 weeks). Embryos were obtained by setting up timed pregnancies. Mice were bred and maintained in the specific pathogen-free animal facilities at Queen Mary University of London or relevant facility. All experiments involving animals were performed in full compliance with UK Home Office regulations and institutional guidelines.

Tissue processing and cell isolation: Isolation of lymphocytes from peripheral lymph nodes (axillary, inguinal and brachial) and spleens involved teasing the organs apart, followed by filtering through an 80μm stainless steel mesh (Sefar Ltd., UK) in fluorescence-activated cell sorting (FACS) buffer [phosphate-buffered saline (PBS) containing 2% heat-inactivated foetal calf serum (FCS; Invitrogen) and 5mM ethylenediaminetetraacetic acid (EDTA; Invitrogen)], to obtain single-cell suspensions. Erythrocytes were osmotically lysed in ACK lysis buffer (Invitrogen) and cells washed twice in FACS buffer. Single-cell suspensions of foetal, neonatal and post-natal thymocytes were obtained by gently homogenizing thymic lobes followed by straining through 40μm strainers (BD).

Foetal Thymic Organ Cultures (FTOC): E14 or E15 thymic lobes from B6 mice were cultured on nucleopore membrane filter discs (Whatman) in FTOC medium (RPMI-1640 with 10% FCS, 1% penicillin and streptomycin, 50μM β-mercaptoethanol, and 2mM L-glutamine) for 7-14 days (unless otherwise indicated). In some experiments anti-TCRδ antibody (GL3; 1μg/ml unless otherwise indicated), anti-CD3ε antibody (2C11; 1 μg/ml unless otherwise indicated), or MEK1/2 inhibitor UO126 (5μM; Sigma-Aldrich) were added to the cultures. Cultures containing antibody or inhibitor were rested overnight in fresh FTOC medium before analysis. All thymic organ cultures were subsequently analysed by flow cytometry.

In vivo treatment with monoclonal anti-CD3ε antibody: Time-mated pregnant WT mice at 13-days post-conception were injected intraperitoneally (i.p.) with anti-CD3ε antibody (2C11; 40 μg in 500 μl PBS per mouse) or PBS only, and allowed to give birth. Thymocytes were isolated from 2-day old neonatal mice as described above, and processed for analysis by flow cytometry.

Cell sorting and OP9-DL1 co-cultures: The OP9-DL1 cell line was provided by J.C Zúniga-Pflücker (University of Toronto, Canada) (Schmitt and Zuniga-Pflucker, 2002). OP9-DL1 cells were maintained in Dulbecco's modified Eagles's medium (DMEM) with GlutaMAX (Invitrogen), 10% FCS, 1% penicillin and streptomycin, 50 μM β-mercaptoethanol (Invitrogen), and 1% non-essential amino acids (Invitrogen) at 37°C and 5% CO_2. OP9-DL1 cells were passaged 1:4 or 1:5 every 2 days and were maintained at 75% confluency. Thymocytes from B6 E15 thymic lobes cultured for 7 days were isolated (as described above), stained and sorted under sterile conditions on a FACSAria (BD) into the four mature γδ$^{24(-)}$ subsets i.e. CD44$^{(-)}$CD45RB$^{(-)}$ "a", CD44$^{(-)}$CD45RB$^{(+)}$ "b", CD44$^{(+)}$CD45RB$^{(+)}$ "c", and CD44$^{(hi)}$CD45RB$^{(-)}$ "d". Sorted cells were seeded in triplicate onto a semi-confluent monolayer of OP9-DL1 cells cultured in flat-bottom 96-well plates with 200 μl of OP9-DL1 medium supplemented with 5 ng/ml Flt3 ligand (Miltenyi Biotec) and 1 ng/ml interleukin-7 (IL-7; Miltenyi Biotec). IL-7 and Flt3 ligand were replenished every 3 days. Cells were incubated for 5 days at 37°C and 5% CO_2. Cultured cells were subsequently analysed by flow cytometry.

Flow cytometry: Fluorochrome-conjugated antibodies (purchased from eBioscience, BD or Biolegend) against the following cell surface molecules and cytokines were used: CCR6 (29-2L17), CD3ε (145-2C11), CD24 (M1/69), CD25 (PC61), CD27 (LG.7F9), CD44 (IM7), CD45RB (C363.16A), CD73 (TY/11.8), CD122 (TM-b1), CD127 (IL-7R; SB/199), NK1.1 (PK136), TCRδ (GL3), Vγ1 (2.11), Vγ4 (UC3-10A6), Vγ5 (536), Vγ7 (F2.67; kindly provided by Dr Pablo Pereira), IFNγ (XMG1.2), IL-4 (11B11) and IL-17A (eBio17B7). 17D1 antibody (rat IgM) that recognises Vγ5Vδ1 and Vγ6Vδ1 was kindly provided by Prof Adrian Hayday as hybridoma supernatant which is used as the primary staining reagent, followed by staining with fluorochrome-conjugated anti-rat IgM antibody. For cell surface staining, thymocytes and lymphocytes were incubated on ice with anti-CD16/CD32 (2.4G2; eBioscience) to block Fc receptors and stained with fluorochrome-conjugated antibodies diluted in FACS buffer. For detection of both Vγ5Vδ1 and Vγ6Vδ1, cells were pre-stained with anti-TCRδ (GL3) antibody at room temperature followed by staining with 17D1. After staining, cells were washed and re-suspended in FACS buffer containing 0.5μg/ml DAPI (Invitrogen) for dead cell exclusion prior to analysis. For intracellular cytokine staining, cells were

stimulated with 50ng/ml phorbol 12-myristate 13-acetate (PMA; Sigma) and 1μg/ml ionomycin (Sigma) for 4h (or 18h for IL-4 detection) at 37°C; 10μg/ml Brefeldin A (eBioscience) and 2μM Monensin (eBioscience) were added during the last 2h (or 4h for IL-4 detection). Cells were stained for cell surface markers and Zombie Aqua™ Fixable Viability dye (Biolegend) for dead cell exclusion, fixed with IC fixation buffer (eBioscience) for 15min on ice and subsequently permeabilised and stained with intracellular cytokine-specific antibodies diluted in permeabilisation buffer (eBioscience). For intracellular detection of transcription factors, cells were fixed and permeabilised with the Foxp3/Transcription Factor buffer set (eBioscience) as per manufacturer's instructions and subsequently stained with antibody to RORγt (B2D; eBioscience) and T-bet (4B10; eBioscience). For detection of apoptosis, cells were stained with Annexin V (Biolegend) followed by the addition of DAPI (0.05 μg/ml) prior to sample acquisition. Samples were acquired using an LSR-II flow cytometer (BD) or Canto II (BD) and data were analysed using FlowJo software (Tree Star, Inc.).

Statistical analysis: Data are presented as mean ± s.d. Student's *t*-test or one-way analysis of variance (ANOVA) were used to assess statistical significance of differences between groups. A difference was considered significant if $P \leq 0.05$.

References:

Schmitt, T.M., and Zuniga-Pflucker, J.C. (2002). Induction of T cell development from hematopoietic progenitor cells by delta-like-1 *in vitro*. Immunity *17*, 749-756.
