# Supplementary material for: Strong TCRγδ Signaling Prohibits Thymic Development of IL-17A-Secreting γδ T Cells
Source: Cell Rep. 2017 Jun 20;19(12):2469–76. doi: 10.1016/j.celrep.2017.05.071 (PMC5489697; doi:10.1016/j.celrep.2017.05.071)
Supplement: Document S2. Article plus Supplemental Information [file mmc2.pdf]

Report

Cell Reports

Strong TCRγδ Signaling Prohibits Thymic Development of IL-17A-Secreting γδ T Cells

Graphical Abstract

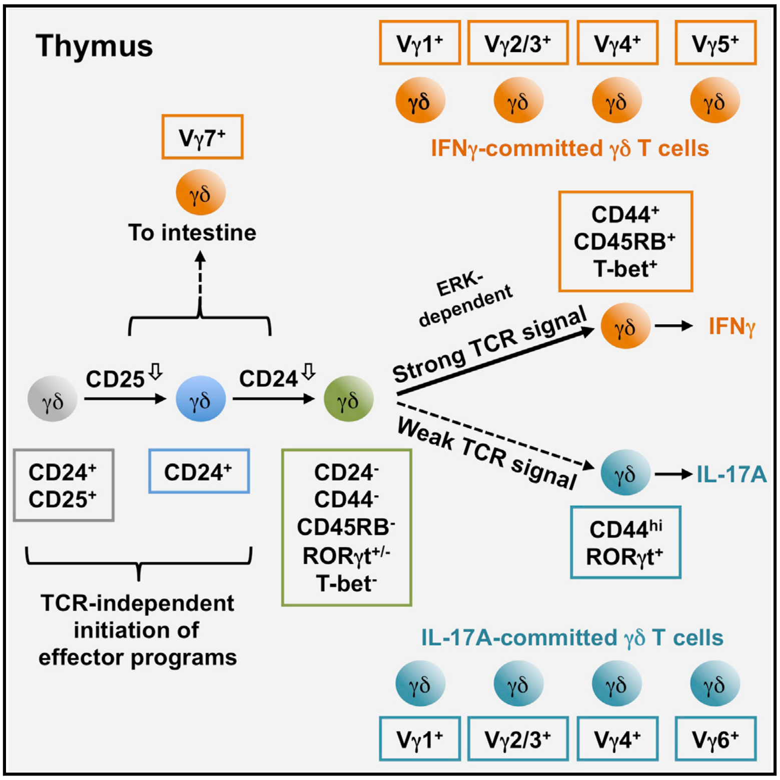

Highlights

- CD44 and CD45RB identify two distinct thymic γδ T cell developmental pathways
- Cytokine-secretion-independent identification of effector fate-committed γδ T cells
- Sizable numbers of IL-17A-committed γδ T cells express Vγ1 and Vγ2/3 chains
- Increased TCRγδ signal strength prohibits development of IL-17A-secreting γδ T cells

Authors

Nital Sumaria, Capucine L. Grandjean, Bruno Silva-Santos, Daniel J. Pennington

Correspondence

d.pennington@qmul.ac.uk

In Brief

Sumaria et al. identify distinct thymic pathways that generate murine γδ T cells pre-committed to the secretion of IL-17A or IFNγ. This permits assessment of TCRγδ signal strength in thymic commitment to γδ T cell effector fate; increased TCRγδ signal strength profoundly prohibits development of all IL-17A-secreting γδ T cells.

Sumaria et al., 2017, Cell Reports *19*, 2469–2476
June 20, 2017 © 2017 The Authors.
http://dx.doi.org/10.1016/j.celrep.2017.05.071

Cell Reports
Report

OPEN ACCESS
CellPress

Strong TCRγδ Signaling Prohibits Thymic Development of IL-17A-Secreting γδ T Cells

Nital Sumaria,[1] Capucine L. Grandjean,[1] Bruno Silva-Santos,[2] and Daniel J. Pennington[1,3,*]
[1]Blizard Institute, Barts and The London School of Medicine, Queen Mary University of London, 4 Newark Street, London E1 2AT, UK
[2]Instituto de Medicina Molecular, Faculdade de Medicina, Universidade de Lisboa, 1600-276 Lisboa, Portugal
[3]Lead Contact
*Correspondence: d.pennington@qmul.ac.uk
http://dx.doi.org/10.1016/j.celrep.2017.05.071

SUMMARY

Despite a growing appreciation of γδ T cell contributions to numerous immune responses, the mechanisms that underpin their thymic development remain poorly understood. Here, using precursor/product relationships, we identify thymic stages in two distinct developmental pathways that generate γδ T cells pre-committed to subsequent secretion of either IL-17A or IFNγ. Importantly, this framework for tracking γδ T cell development has permitted definitive assessment of TCRγδ signal strength in commitment to γδ T cell effector fate; increased TCRγδ signal strength profoundly prohibited the development of all IL-17A-secreting γδ T cells, regardless of Vγ usage, but promoted the development of γδ progenitors along the IFNγ pathway. This clarifies the recently debated role of TCRγδ signal strength in commitment to distinct γδ T cell effector fates and proposes an alternate methodology for the study of γδ T cell development.

INTRODUCTION

γδ T cells make rapid non-redundant contributions in numerous disease settings that include malaria (Behr et al., 1996) and tuberculosis infections (Kabelitz et al., 1991), as well as immunopathologies such as psoriasis (Laggner et al., 2011). In addition, γδ T cells display potent anti-tumor capabilities, such that a tumor-associated γδ T cell expression signature was the most favorable immune-related positive prognostic indicator in analyses of more than 18,000 tumors (Gentles et al., 2015).

Murine γδ T cells execute their effector capacities through provision of cytokines (Pang et al., 2012). Anti-tumor function is associated with IFNγ production (Gao et al., 2003), whereas IL-17A drives γδ T cell responses to extracellular bacteria and fungi (Dejima et al., 2011; Hamada et al., 2008). This delivery of IFNγ or IL-17A mirrors that of αβ T helper cell clones that acquire cytokine-secreting functions only at the point of peripheral activation in secondary lymphoid tissue. By contrast, γδ T cells largely acquire their effector potential (to secrete IFNγ or IL-17A) in the thymus, well before their participation in subsequent immune responses (Ribot et al., 2009).

The mechanisms that drive thymic commitment to γδ T cell effector function are still unclear. "Strong" ligand-dependent signaling through the γδ T cell receptor (TCRγδ) was suggested to promote commitment to an IFNγ-secreting fate (Jensen et al., 2008; Muñoz-Ruiz et al., 2016; Turchinovich and Hayday, 2011), with weaker, possibly ligand-independent TCR signaling being required for IL-17A production (Jensen et al., 2008; Turchinovich and Hayday, 2011). However, recent studies have also implicated "strong" TCRγδ signals in commitment to an IL-17A-secreting fate (Coffey et al., 2014; Wencker et al., 2014). Alternatively, evidence exists for TCR-independent commitment to effector potentials. For example, IL-17A-secreting γδ T cells develop exclusively in a perinatal window, such that adoptive transfer of adult bone marrow will not reconstitute the IL-17A-secreting γδ T cell compartment (Haas et al., 2012). IL-17A-producing γδ T cells are also suggested to preferentially develop from $CD4^{-}CD8^{-}$ double-negative (DN) 2 cells (rather than DN3 cells) (Shibata et al., 2014). And certain γδ T cell subsets (e.g., those using a TCRγ chain incorporating variable region 4; $V\gamma4^{+}$ cells) may inherently require certain transcription factors (e.g., Sox-13) (Gray et al., 2013; Malhotra et al., 2013). Clearly, a better understanding of γδ T cell development is required that will provide critical insight into γδ T cell biology.

There is presently no accepted approach for stage-wise assessment of thymic γδ T cell development. Indeed, although studies have analyzed Vγ usage (Gray et al., 2013; Turchinovich and Hayday, 2011), acquisition of effector potential (Jensen et al., 2008; Lombes et al., 2015; Ribot et al., 2009; Turchinovich and Hayday, 2011), gene transcription (Schmolka et al., 2013), and surface marker expression (Coffey et al., 2014; Haas et al., 2009; Jensen et al., 2008; Lombes et al., 2015; Ribot et al., 2009; Turchinovich and Hayday, 2011), a methodology that combines these parameters, akin to that for αβ T cells, is still lacking. Here, using precursor/product relationships, we identify thymic stages in two distinct developmental pathways that generate γδ T cells committed to subsequent secretion of IL-17A or IFNγ. This exposes a temporal disconnect between thymic commitment to effector fate and immediate capacity to display effector function. Cytokine-independent identification of fate-committed γδ T cells reveals the full contribution of Vγ-chain-expressing progenitors to both cytokine-producing pathways through ontogeny, highlighting sizable numbers of IL-17A-committed cells expressing Vγ1 and Vγ2/3 chains. Importantly, these analyses also permit definitive assessment of TCRγδ signal strength in commitment to γδ T cell effector fate; increased TCRγδ signal strength profoundly

CrossMark
This is an open access article under the CC BY license (http://creativecommons.org/licenses/by/4.0/).

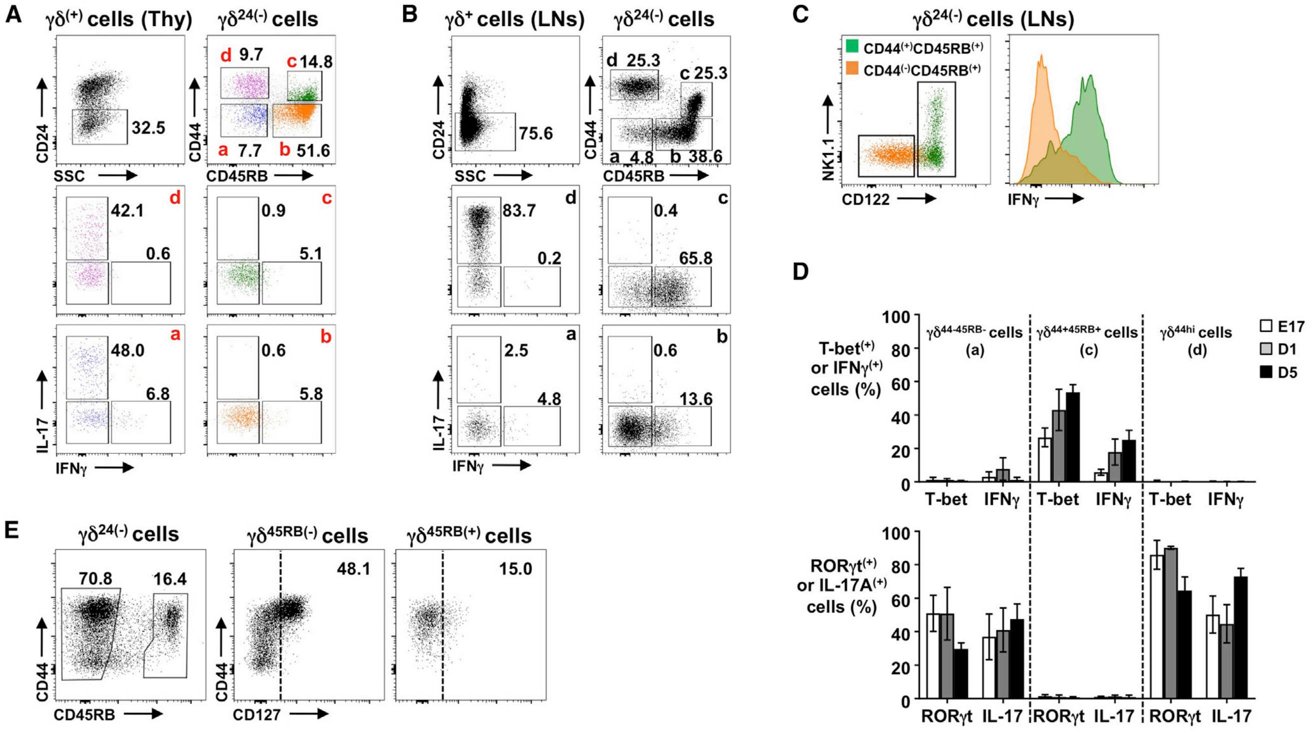

Figure 1. CD24, CD44, and CD45RB Identify Functionally Distinct γδ T Cell Subsets

(A and B) γδ T cells from (E17) thymic lobes (A) or adult lymph nodes (LNs) (B). For both, CD24$^-$ γδ T cells (TCRδ$^+$CD3ε$^+$) from top left are sub-divided by CD44 and CD45RB (top right; subsets a–d). Middle and bottom plots show intracellular (i.c.) staining for IL-17A/IFNγ in subsets a–d.

(C) CD44$^-$CD45RB$^+$ b (orange) and CD44$^+$CD45RB$^+$ c (green) γδ T cells from LNs overlaid to show CD122/NK1.1 (left) and i.c. IFNγ (right).

(D) Summary of T-bet/IFNγ (top) or RORγt/IL-17A (bottom) in CD44$^-$CD45RB$^-$, CD44$^+$CD45RB$^+$, and CD44^{hi}CD45RB$^-$ thymic γδ T cells through ontogeny. For cytokines, cells were stimulated 4 hr ex vivo with PMA/ionomycin.

(E) CD127 on CD45RB$^-$ (middle) and CD45RB$^+$ (right) thymic γδ T cells from neonatal mice.

Data are representative of at least two independent experiments (A–C and E; n ≥ 6 mice), and (D; n ≥ 4 mice or ≥4 lobes pooled for E17). Summarized data are represented as mean ± SD. See also Figures S1 and S2.

prohibits the development of all IL-17A-secreting γδ T cells, regardless of Vγ usage but promoted the development of γδ progenitors along the IFNγ pathway. These observations provide important insights into functional γδ T cell biology.

RESULTS

CD24, CD44, and CD45RB Identify Functionally Distinct γδ T Cell Subsets

There is no consensus for describing stages in murine γδ T cell development. Thus, we re-assessed, on perinatal, neonatal, and post-natal thymic γδ T cells, the expression of γδ T cell surface markers (Coffey et al., 2014; Haas et al., 2009; Jensen et al., 2008; Ribot et al., 2009; Wencker et al., 2014) combined with intracellular (i.c.) staining for IFNγ and IL-17A (Figure S1). This revealed that staining for CD24, CD44, and CD45RB neatly segregated both thymic (Figure 1A) and peripheral (Figure 1B) γδ T cells, throughout ontogeny (Figure S2A), into two apparent "pathways"; CD24$^-$ cells that expressed high CD44 but not CD45RB were committed to IL-17A secretion, but did not make IFNγ, whereas cells that had upregulated CD45RB had potential to secrete IFNγ but not IL-17A (Figures 1A and 1B). CD45RB^{hi} γδ T cells can also upregulate CD44, which correlates with NK1.1 and CD122 expression and robust peripheral commitment to IFNγ secretion (Figure 1C). Consistent with IL-17A-secreting potential (Michel et al., 2012; Ribot et al., 2009; Schmolka et al., 2013), CD44^{hi}CD45RB$^-$ γδ T cells were RORγt$^+$T-bet^{lo} (Figures 1D and S2B) and expressed significant CD127 (the IL-7Rα chain) that appeared to follow upregulation of CD44 (Figure 1E). By contrast, CD44$^+$CD45RB$^+$ γδ cells were T-bet$^+$RORγt^{lo} and displayed little CD127 (Figures 1D, 1E, and S2B). Finally, although we could not detect IL-4-secreting γδ T cells directly ex vivo, a small fraction of the CD44$^+$CD45RB$^+$ subset from both post-natal thymus and adult spleen produced IL-4 after 18 hr culture in PMA/ionomycin (Figure S2C). Thus, in the thymus and periphery, CD24, CD44, and CD45RB neatly segregate γδ T cells into subsets with IL-17A- or IFNγ-secreting potential.

γδ T Cell Commitment to Cytokine-Secreting Potential Follows One of Two Pathways

CD44 and CD45RB appear to segregate CD24$^-$ γδ T cells into two developmental pathways, whereby CD44$^-$CD45RB$^-$ cells develop as either CD44^{hi}CD45RB$^-$ IL-17A-committed γδ

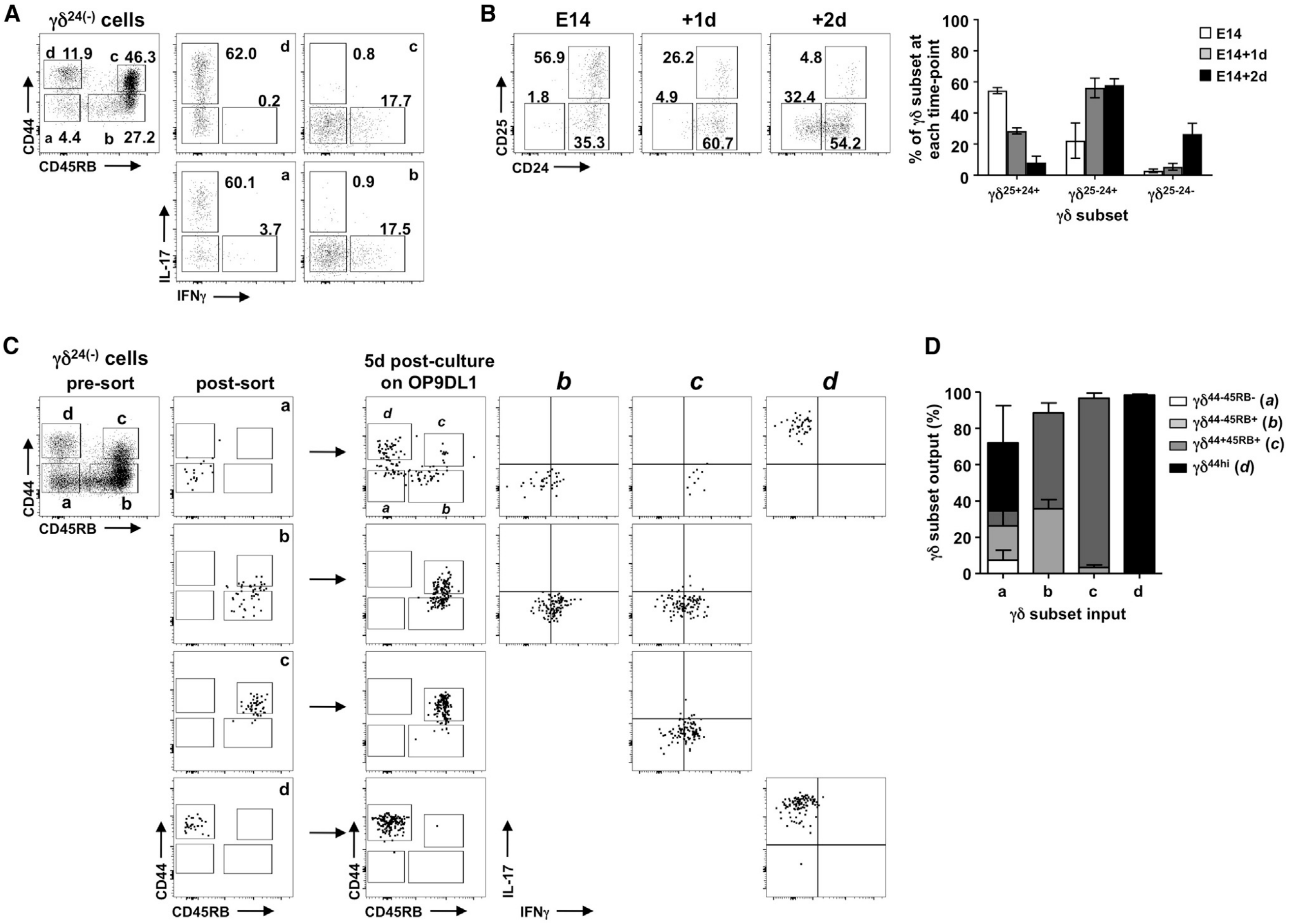

Figure 2. γδ T Cell Commitment to Cytokine-Secreting Potential Follows One of Two Pathways

(A) Left shows CD44/CD45RB on CD24⁻ γδ T cells from E15 lobes after 7-day FTOC. Right shows i.c. IL-17A/IFNγ in subsets a–d.

(B) CD25/CD24 on γδ T cells from E14 lobes ex vivo (left plot) or after 1- or 2-day FTOC (right plots). Summary data are shown to right of plots.

(C) Cells sorted from E15 7-day FTOC; a–d (left plot), with post-sort re-runs. Sorted cells from 5-day OP9-DL1 cultures were then stained for CD44/CD45RB and i.c. IL-17A/IFNγ.

(D) Summary of (C) showing recovered cells (output) after 5-day culture of sorted cells (input).

Data are representative of at least two independent experiments. Summarized data are represented as mean ± SD.

T cells or CD45RB^{+} IFNγ-committed γδ T cells. To formally investigate this hypothesis, we used fetal thymic organ culture (FTOC) that re-capitulates thymic T cell development in vitro and is suited to studying γδ T cell development that occurs predominantly in the perinatal period. Indeed, E15 thymic lobes cultured for 7 days generate γδ T cell subsets similar to those observed ex vivo (Figure 2A). To show precursor/product relationships, we first took E14 lobes and cultured them in FTOC for either 1 or 2 days. Ex vivo, γδ T cells from E14 lobes are all CD24^{+}, with a sizable proportion also CD25^{+} (Figure 2B). Consistent with CD25^{+} γδ T cells' being the earliest γδ T cell subset in the thymus (Prinz et al., 2006; Ribot et al., 2009), the proportion of these cells is notably reduced over a 2-day culture period. On day 1, CD24^{+}CD25^{-} cells were the dominate subset, whereas by day 2, a substantial proportion of cells became CD24^{-}; this suggests a developmental progression from CD25^{+}CD24^{+} to CD25^{-}CD24^{+} to CD25^{-}CD24^{-}.

We next fluorescence-activated cell sorting (FACS)-purified the four CD24^{-} γδ T cell populations from 7-day FTOC of E15 thymic lobes. These were CD44^{-}CD45RB^{-} a cells, CD44^{-}CD45RB^{+} b cells, CD44^{+}CD45RB^{+} c cells, and CD44^{hi}CD45RB^{-} d cells (Figure 2C). Sorted cells were then cultured for a further 5 days on OP9-DL1 stromal cells, which also support thymic T cell development, and subsequently re-assessed. On re-analysis, both CD44^{+}CD45RB^{+} and CD44^{hi}CD45RB^{-} subsets displayed characteristics of terminally differentiated cells, retaining both their CD44/CD45RB expression and complete and full commitment to IFNγ- and IL-17A-secreting potential, respectively (Figures 2C and 2D). In contrast, the CD44^{-}CD45RB^{-} subset differentiated to all other phenotypes, with their CD45RB^{+} products displaying expected IFNγ-secreting potential and their CD44^{hi}CD45RB^{-} products appearing committed to IL-17A. Finally, CD44^{-}CD45RB^{+} cells gave rise to a significant number of CD44^{+}CD45RB^{+} products (Figures 2C and 2D), suggesting a developmental pathway

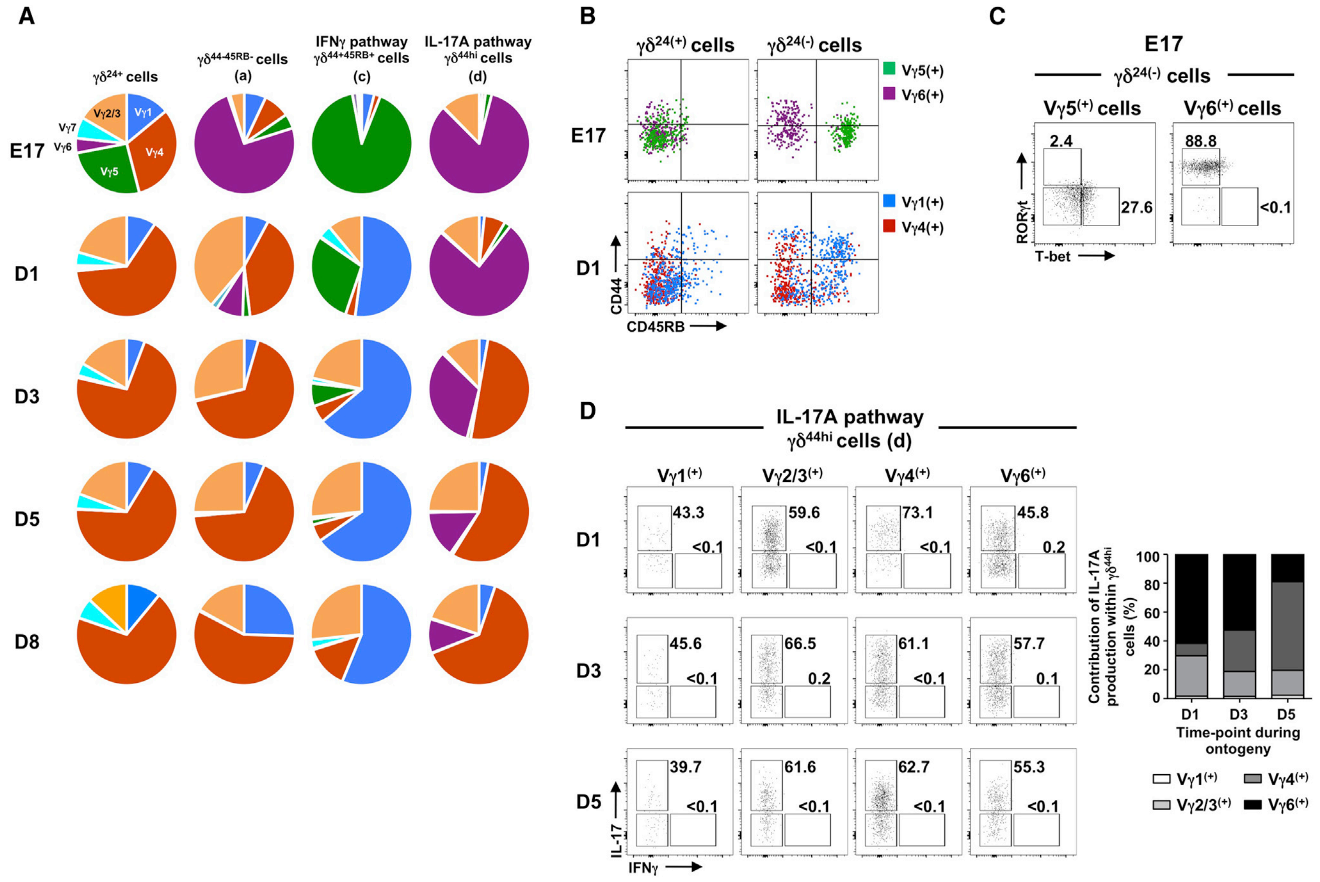

Figure 3. $V\gamma5^+$ and $V\gamma6^+$ Cells Segregate to One of Two Developmental Pathways

(A) Vγ usage by $CD24^+$, $CD44^-CD45RB^-$, $CD44^+CD45RB^+$, and $CD44^{hi}CD45RB^-$ thymic γδ T cells through ontogeny.

(B) CD44/CD45RB on $V\gamma5^+$ (green) and $V\gamma6^+$ ($17D1^+$ purple) cells (top) and $V\gamma1^+$ (blue) and $V\gamma4^+$ (red) cells (bottom) within $CD24^+$ (left) or $CD24^-$ (right) subsets from WT thymus.

(C) i.c. RORγt/T-bet in $V\gamma5^+$ and $V\gamma6^+$ cells within $CD24^-$ γδ pool of E17 thymus.

(D) i.c. IL-17A/IFNγ in thymic Vγ-chain-specific $CD44^{hi}CD45RB^-$ γδ T cells through ontogeny.

Data are representative of at least two (A–C) or one (D) independent experiments. See also Figure S3.

from $CD44^-CD45RB^-$ to $CD44^-CD45RB^+$ to $CD44^+CD45RB^+$ for an IFNγ-secreting fate. Thus, CD24, CD44, and CD45RB identify two distinct γδ T cell development pathways that segregate commitment to either IFNγ- or IL-17A-secreting potential.

$V\gamma5^+$ and $V\gamma6^+$ Cells Fully Segregate to One of the Two Developmental Pathways

The preferential use of γδTCRs that incorporate certain Vγ-regions has been frequently correlated with peripheral cytokine-secreting potential: $V\gamma4^+$ and $V\gamma6^+$ cells being linked to IL-17A production, with $V\gamma1^+$ and $V\gamma5^+$ cells linked to IFNγ (Prinz et al., 2013). However, this is difficult to study in the early thymus, as only a minority of neonatal $CD24^-$ γδ T cells display immediate cytokine-secreting capacity after 4 hr stimulation with PMA/ionomycin (Figure S3A). In contrast, the vast majority of these $CD24^-$ cells have already entered one of the two developmental pathways described above (Figure 1A) and are thus already committed to a cytokine-secreting fate (Figure 2C). To use this extra sensitivity to observe cytokine-committed $TCR\gamma\delta^+$ thymocytes, we assessed through ontogeny, from E17 to day 8 post-birth, Vγ usage of γδ T cells committed to either the IL-17A or IFNγ pathway using staining strategies that detect $V\gamma1^+$, $V\gamma2/3^+$, $V\gamma4^+$, $V\gamma5^+$, $V\gamma6^+$, and $V\gamma7^+$ cells (Figure S3B). Before birth, $V\gamma5^+$ and $V\gamma6^+$ cells dominated the IFNγ-committed and IL-17A-committed pathways, respectively (Figure 3A). Indeed, at E17 almost complete segregation of $V\gamma5^+$ cells to a $CD45RB^+$ fate and $V\gamma6^+$ cells to a $CD44^{hi}CD45RB^-$ fate was observed (Figure 3B), which corresponded to T-bet (but not RORγt) expression in $V\gamma5^+$ cells and RORγt (but not T-bet) expression in $V\gamma6^+$ cells (Figure 3C). However, such precise mapping of Vγ staining to one of the two pathways was not observed for other Vγ regions, as $V\gamma1^+$, $V\gamma2/3^+$, and $V\gamma4^+$ cells were clearly represented in both routes of development (Figures 3A and 3B). Indeed, $V\gamma2/3^+$ cells, which have been overlooked in murine γδ T cell studies to date, make sizable contributions to both pathways and are as capable as either $V\gamma4^+$ or $V\gamma6^+$ (or $V\gamma1^+$) cells of making IL-17A (Figure 3D). Finally, $V\gamma7^+$ cells, which are readily identifiable in early $CD24^+$ subsets, are barely

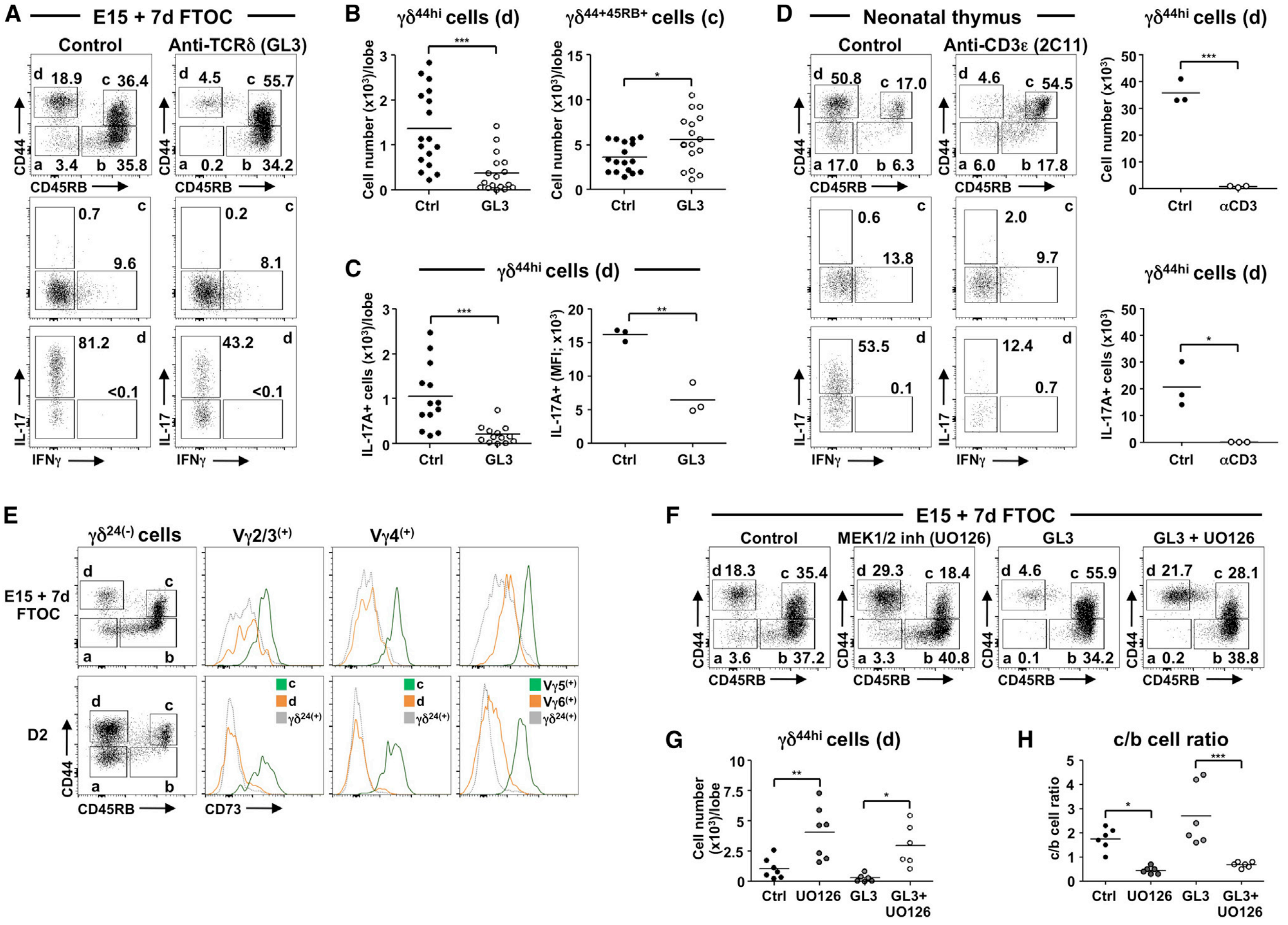

Figure 4. Increased TCRγδ Signal Strength Prohibits Development of IL-17A-Secreting γδ T Cells

(A) CD44/CD45RB (top) on CD24⁻ γδ T cells from E15 7-day FTOC ± GL3 (1 μg/ml). Bottom is i.c. IL-17A/IFNγ in c and d gated from top.

(B) Number of $CD44^{hi}CD45RB^-$ (left) and $CD44^+CD45RB^+$ (right) γδ T cells in 7-day FTOCs ± GL3 described in (A).

(C) Number of $IL\text{-}17A^+$ $CD44^{hi}CD45RB^-$ γδ T cells (left) and MFI of IL-17A in these cells (right) in 7-day FTOCs ± GL3 described in (A).

(D) CD44/CD45RB (top) on thymic $CD24^-$ γδ T cells from neonatal (2-day-old) mice born to time-mated WT mice that received an injection of anti-CD3ε antibody (2C11; 40 μg) or PBS only (control) i.p. Bottom is i.c. IL-17A/IFNγ in c and d gated from top. Graphs show number of $CD44^{hi}CD45RB^-$ (top) and $IL\text{-}17A^+$ $CD44^{hi}CD45RB^-$ (bottom) γδ T cells from neonatal mice described above.

(E) CD44/CD45RB on $CD24^-$ γδ T cells from E15 7-day FTOC (top) or 2-day neonatal thymus (bottom) and CD73 histograms for Vγ-specific $CD44^+CD45RB^+$ (green), $CD44^{hi}CD45RB^-$ (orange), or immature $CD24^+$ (gray) γδ subsets. For $V\gamma5^+$ or $V\gamma6^+$ cells, gray histograms signify all $CD24^+$ γδ T cells.

(F) CD44/CD45RB on $CD24^-$ γδ T cells from E15 7-day FTOC with MEK1/2 inhibitor UO126 (5 μM), GL3 (1 μg/ml), UO126 (5 μM) plus GL3 (1 μg/ml), or control.

(G) Number of $CD44^{hi}CD45RB^-$ γδ T cells in (F).

(H) Ratio of $CD44^+CD45RB^+$ to $CD44^-CD45RB^+$ γδ T cells in (F).

Data points (B, C, G, and H) represent at least four lobes pooled. Data points (D) represent individual neonatal mice. Data are representative of at least three (A–C and F–H), two (E), or one (D) independent experiment. *$p \leq 0.05$, **$p \leq 0.01$, and ***$p \leq 0.001$ (Student's t test or ANOVA). See also Figure S4.

detected in either of the mature $CD24^-$ pathways (Figure 3A), supporting the view that these cells leave the thymus at an early stage of thymic development to seed the murine intestine (Di Marco Barros et al., 2016).

Increased TCRγδ Signal Strength Restricts Development of All IL-17A-Secreting γδ T Cells

The factors that dictate commitment to an IL-17A- or IFNγ-secreting fate are still unclear. Central to this is the role of TCRγδ signaling, as although consensus suggests that "strong" TCRγδ signals favor development of IFNγ-committed cells (Jensen et al., 2008; Muñoz-Ruiz et al., 2016; Turchinovich and Hayday, 2011), conflicting views exist as to the strength of TCRγδ signal required for an IL-17A-secreting fate (Coffey et al., 2014; Jensen et al., 2008; Turchinovich and Hayday, 2011; Wencker et al., 2014). In 7-day FTOC of E15 thymic lobes, addition of anti-TCRγδ antibody GL3, which increases TCRγδ signal strength (Kreslavsky et al., 2008; Turchinovich and Hayday, 2011), clearly reduced the generation of IL-17A-committed cells while significantly increasing the number of $CD44^+CD45RB^+$ cells (Figures 4A and 4B). The effect on those cells capable of immediate IL-17A secretion was particularly dramatic, reducing both absolute

cell number and the amount of IL-17A produced per cell (Figure 4C). This effect was GL3 dose dependent (Figure S4A), was not the result of TCR signaling-induced apoptosis (Figure S4B), and resulted in a complete absence of all Vγ-expressing cells in the IL-17A pathway if GL3 was added to 7- to 14-day FTOC of E14 thymic lobes (Figure S4C). Moreover, intraperitoneal administration to pregnant wild-type (WT) mice at 13-days post-conception of the anti-CD3ε antibody 2C11, which induces similar developmental changes as GL3 in vitro (Figure S4D), also resulted in profound reduction of IL-17A-commited γδ T cells in pups at day 2 after birth (Figure 4D). Finally, and consistent with these findings, cells from the IFNγ pathway, from either 7-day FTOC or day 2 pups ex vivo, displayed significantly more CD73 (a marker linked to [strong] TCRγδ-ligand-induced signaling; Coffey et al., 2014), than cells from the IL-17A pathway, regardless of Vγ usage (Figure 4E).

TCR signals are transduced, in part, by signals through the ERK/MAP kinase cascade (Haks et al., 2005). Hence, to assess the consequences of weaker TCRγδ signaling, the MEK1/2 inhibitor of ERK signaling UO126 was added to 7-day E15 FTOC. Compared with control cultures, UO126 significantly increased cell number in the IL-17A-committed pathway (Figures 4F and 4G) while reducing the ratio of terminally differentiated CD44$^+$CD45RB$^+$ cells to less mature CD44$^-$CD45RB$^+$ cells in the IFNγ-committed pathway (Figure 4H). Importantly, UO126 could also rescue the number of IL-17A-committed cells in FTOC containing GL3 (Figures 4F and 4G) and improved the ratio of CD44$^+$CD45RB$^+$ to CD44$^-$CD45RB$^+$ cells (Figure 4H). Thus, manipulation of TCRγδ signal strength with either crosslinking anti-TCRγδ antibody (stronger) or an ERK pathway inhibitor (weaker) demonstrates that strong TCRγδ signals are prohibitive for the generation of γδ T cells destined to secrete IL-17A, regardless of the Vγ chain they use.

DISCUSSION

Here, we describe a straightforward methodology to study the sequential thymic development of murine γδ T cells. TCRδ$^+$CD25$^+$ cells, which are considered the earliest thymic γδ T cell subset (Prinz et al., 2006; Ribot et al., 2009), begin development by downregulating CD25, followed by CD24. How this is triggered remains to be elucidated, but TCRγδ signaling was shown to be necessary to pass beyond a TCRγδ^{lo}CD25$^+$ stage (Prinz et al., 2006). When cultured as a population, CD24$^-$ γδ thymocytes that are CD44$^-$CD45RB$^-$ give rise to either IL-17A-committed CD44^{hi}CD45RB$^-$ cells that express RORγt but not T-bet or to IFNγ-committed CD45RB$^+$ cells that (more gradually) express T-bet but not RORγt. Interestingly, our CD44/CD45RB plots show overlap with CD44/Ly-6C plots suggested to identify naive-like and memory-like peripheral γδ T cell subsets (Lombes et al., 2015). Thus, combination staining of CD44 with both Ly-6C and CD45RB may prove particularly insightful.

Importantly, our analyses identify two thymic pathways of functional γδ T cell differentiation that diverge from a common CD24$^-$CD44$^-$CD45RB$^-$ phenotype. Whether each CD24$^-$CD44$^-$CD45RB$^-$ cell has potential to enter both pathways, or whether the subset instead contains both IL-17A- and IFNγ-committed progenitors, is still uncertain. However, that some CD24$^-$CD44$^-$CD45RB$^-$ γδ T cells can already make either IL-17A or IFNγ (but not both) supports a model in which commitment to an IL-17A- or IFNγ-secreting fate, with initial expression of corresponding "master" transcriptional regulators (Malhotra et al., 2013), spans an early window of development that includes CD24$^+$ subsets. Nonetheless, commitment appears fully established by the time cells upregulate either CD44 or CD45RB from the CD24$^-$CD44$^-$CD45RB$^-$ stage. Notably, these committed cells do not necessarily display immediate capacity to secrete cytokine. This is particularly evident for CD45RB$^+$ cells in the IFNγ pathway as only a minority secrete IFNγ ex vivo. However, when isolated and cultured on OP9-DL1 cells for a further 5 days, virtually all then secrete IFNγ (but not IL-17A). These observations suggest thymic commitment of γδ progenitors to distinct effector fates is distinguishable (temporally and presumably mechanistically) from actual capacity to secrete cytokine.

The identification of surface marker-defined, cytokine secretion-independent developmental pathways for γδ T cell generation facilitated re-examination of TCRγδ signal strength requirements for thymic commitment of γδ progenitors to specific effector fates. Strong antibody-induced TCRγδ signaling favored the IFNγ pathway (Jensen et al., 2008; Muñoz-Ruiz et al., 2016; Ribot et al., 2009; Turchinovich and Hayday, 2011). This was consistent with significantly higher expression of CD73 (recently purported to reflect increased TCRγδ signaling; Coffey et al., 2014) on cells committed to secrete IFNγ compared with those in the IL-17A pathway. Cells in the IFNγ pathway express CD45RB that is upregulated on developing Vγ5$^+$Vδ1$^+$ cells in the presence of Skint1 (Turchinovich and Hayday, 2011), a possible ligand for the Vγ5Vδ1 TCR (Barbee et al., 2011). In the absence of Skint1, Vγ5$^+$ cells instead adopt characteristics of Vγ6$^+$ cells, including capacity to secrete IL-17A (Turchinovich and Hayday, 2011). In our studies, strong antibody-induced TCRγδ signaling prevented the development of all cells destined for the IL-17A pathway, which included a sizable number of Vγ1$^+$ and Vγ2/3$^+$ cells, as well as Vγ4$^+$ and Vγ6$^+$ cells. This appears at odds with a recent report that revealed an absence of IL-17A-committed (but not IFNγ-committed) γδ T cells in SKG mice that have severely reduced Zap-70 activity (Wencker et al., 2014). Although interpreted as showing that strong TCRγδ signaling is required for commitment to an IL-17A-secreting fate, we instead prefer the explanation that generation of IL-17A-producing γδ T cells is simply Zap-70 (and/or Syk) dependent. Importantly however, our data show that this Zap-70 dependence cannot equate to transducing a strong TCRγδ signal.

Our results indicate that at least one downstream mediator of strong TCRγδ signaling is the ERK/MAP kinase pathway, as its inhibition promoted the IL-17A pathway while reducing progression through the IFNγ pathway. Moreover, it reversed many (but not all) effects of increased TCRγδ signal strength mediated by anti-TCRδ antibody. Thus, activation of the ERK/MAP kinase pathway by strong TCRγδ signaling is a key limiter of progression to an IL-17A-secreting fate. As mentioned above, such strong signaling may reflect engagement of TCR ligand, as supported by complete segregation, in the prenatal thymus, of Vγ5$^+$ cells to the IFNγ pathway and Vγ6$^+$ cells to the IL-17A pathway (Barbee et al., 2011; Turchinovich and Hayday, 2011). However,

$\gamma\delta$ T cells bearing $V\gamma1^+$, $V\gamma2/3^+$, or $V\gamma4^+$ TCRs were readily detected in both pathways. This could imply that only some of these TCRs engage ligand. Alternatively, ligand-independent signaling (Mahtani-Patching et al., 2011) that depends on surface expression levels and/or features of particular $V\gamma$ regions may dictate the proportion of cells that successfully engage the ERK/MAP kinase pathway. Finally, $V\gamma7^+$ cells, which largely seed the murine intestine, are not present in either pathway, suggesting that factors other than TCR$\gamma\delta$ signaling should also be considered (Di Marco Barros et al., 2016). These ideas, and the involvement of other downstream signaling cascades, are currently under investigation.

EXPERIMENTAL PROCEDURES

Additional details are available in Supplemental Experimental Procedures.

Mice

C57BL/6 (B6) mice were purchased from Charles River Laboratories. All mice were fetal (E14–E17), neonatal (1–3 days), post-natal (4–8 days), or adult (8–12 weeks; female). All experiments involving animals were performed in full compliance with UK Home Office regulations and institutional guidelines.

FTOCs

Thymic lobes from B6 mice were cultured on Nuclepore membrane filter discs (Whatman) in complete RPMI-1640 medium plus 10% fetal calf serum (FCS) for 7–14 days.

OP9-DL1 Co-cultures

OP9-DL1 cells were provided by J.C. Zúniga-Pflücker (University of Toronto).

Flow Cytometry

For detection of $V\gamma5V\delta1$ and $V\gamma6V\delta1$, cells were pre-stained with GL3 followed by 17D1. For i.c. cytokine staining (eBioscience), cells were stimulated with 50 ng/ml phorbol 12-myristate 13-acetate (PMA; Sigma) and 1 μg/ml ionomycin (Sigma) for 4 hr at 37°C. Acquisition was performed with an LSR-II or a Canto II (BD). Analysis was performed using FlowJo (Tree Star).

Statistical Analysis

GraphPad Prism software was used to analyze data, which are presented as mean ± SD. Two-tailed Student's unpaired t test was used when only two groups were compared, and one-way ANOVA with Tukey's test was used for multiple comparisons. Significance was determined at $p \leq 0.05$.

SUPPLEMENTAL INFORMATION

Supplemental Information includes Supplemental Experimental Procedures and four figures and can be found with this article online at http://dx.doi.org/10.1016/j.celrep.2017.05.071.

AUTHOR CONTRIBUTIONS

N.S. and C.L.G. performed experiments. N.S. and D.J.P. analyzed the data. B.S.-S. and D.J.P. designed the study. D.J.P. and N.S. wrote the paper.

ACKNOWLEDGMENTS

We thank J. Pang, J. Neves, M. Rei, S. Martin, K. Stoenchev, M. Muñoz-Ruiz, J. Ribot, and V. Sofra for help and advice; G. Turchinovich and A. Hayday for insightful discussions; and our BSU and flow facilities for technical assistance. This work was supported by grants from the European Research Council (StG_260352 to B.S.-S.) and the Wellcome Trust (092973/Z/10/Z to D.J.P.).

Received: February 12, 2017
Revised: May 2, 2017
Accepted: May 22, 2017
Published: June 20, 2017

REFERENCES

Barbee, S.D., Woodward, M.J., Turchinovich, G., Mention, J.J., Lewis, J.M., Boyden, L.M., Lifton, R.P., Tigelaar, R., and Hayday, A.C. (2011). Skint-1 is a highly specific, unique selecting component for epidermal T cells. Proc. Natl. Acad. Sci. U S A *108*, 3330–3335.

Behr, C., Poupot, R., Peyrat, M.A., Poquet, Y., Constant, P., Dubois, P., Bonneville, M., and Fournie, J.J. (1996). Plasmodium falciparum stimuli for human gammadelta T cells are related to phosphorylated antigens of mycobacteria. Infect. Immun. *64*, 2892–2896.

Coffey, F., Lee, S.Y., Buus, T.B., Lauritsen, J.P., Wong, G.W., Joachims, M.L., Thompson, L.F., Zúñiga-Pflücker, J.C., Kappes, D.J., and Wiest, D.L. (2014). The TCR ligand-inducible expression of CD73 marks $\gamma\delta$ lineage commitment and a metastable intermediate in effector specification. J. Exp. Med. *211*, 329–343.

Dejima, T., Shibata, K., Yamada, H., Hara, H., Iwakura, Y., Naito, S., and Yoshikai, Y. (2011). Protective role of naturally occurring interleukin-17A-producing $\gamma\delta$ T cells in the lung at the early stage of systemic candidiasis in mice. Infect. Immun. *79*, 4503–4510.

Di Marco Barros, R., Roberts, N.A., Dart, R.J., Vantourout, P., Jandke, A., Nussbaumer, O., Deban, L., Cipolat, S., Hart, R., Iannitto, M.L., et al. (2016). Epithelia use butyrophilin-like molecules to shape organ-specific $\gamma\delta$ T cell compartments. Cell *167*, 203–218.e17.

Gao, Y., Yang, W., Pan, M., Scully, E., Girardi, M., Augenlicht, L.H., Craft, J., and Yin, Z. (2003). Gamma delta T cells provide an early source of interferon gamma in tumor immunity. J. Exp. Med. *198*, 433–442.

Gentles, A.J., Newman, A.M., Liu, C.L., Bratman, S.V., Feng, W., Kim, D., Nair, V.S., Xu, Y., Khuong, A., Hoang, C.D., et al. (2015). The prognostic landscape of genes and infiltrating immune cells across human cancers. Nat. Med. *21*, 938–945.

Gray, E.E., Ramírez-Valle, F., Xu, Y., Wu, S., Wu, Z., Karjalainen, K.E., and Cyster, J.G. (2013). Deficiency in IL-17-committed $V\gamma4(+)$ $\gamma\delta$ T cells in a spontaneous Sox13-mutant CD45.1(+) congenic mouse substrain provides protection from dermatitis. Nat. Immunol. *14*, 584–592.

Haas, J.D., González, F.H., Schmitz, S., Chennupati, V., Föhse, L., Kremmer, E., Förster, R., and Prinz, I. (2009). CCR6 and NK1.1 distinguish between IL-17A and IFN-gamma-producing gammadelta effector T cells. Eur. J. Immunol. *39*, 3488–3497.

Haas, J.D., Ravens, S., Düber, S., Sandrock, I., Oberdörfer, L., Kashani, E., Chennupati, V., Föhse, L., Naumann, R., Weiss, S., et al. (2012). Development of interleukin-17-producing $\gamma\delta$ T cells is restricted to a functional embryonic wave. Immunity *37*, 48–59.

Haks, M.C., Lefebvre, J.M., Lauritsen, J.P., Carleton, M., Rhodes, M., Miyazaki, T., Kappes, D.J., and Wiest, D.L. (2005). Attenuation of gammadeltaTCR signaling efficiently diverts thymocytes to the alphabeta lineage. Immunity *22*, 595–606.

Hamada, S., Umemura, M., Shiono, T., Tanaka, K., Yahagi, A., Begum, M.D., Oshiro, K., Okamoto, Y., Watanabe, H., Kawakami, K., et al. (2008). IL-17A produced by gammadelta T cells plays a critical role in innate immunity against listeria monocytogenes infection in the liver. J. Immunol. *181*, 3456–3463.

Jensen, K.D., Su, X., Shin, S., Li, L., Youssef, S., Yamasaki, S., Steinman, L., Saito, T., Locksley, R.M., Davis, M.M., et al. (2008). Thymic selection determines gammadelta T cell effector fate: antigen-naive cells make interleukin-17 and antigen-experienced cells make interferon gamma. Immunity *29*, 90–100.

Kabelitz, D., Bender, A., Prospero, T., Wesselborg, S., Janssen, O., and Pechhold, K. (1991). The primary response of human gamma/delta + T cells to Mycobacterium tuberculosis is restricted to V gamma 9-bearing cells. J. Exp. Med. *173*, 1331–1338.

Kreslavsky, T., Garbe, A.I., Krueger, A., and von Boehmer, H. (2008). T cell receptor-instructed alphabeta versus gammadelta lineage commitment revealed by single-cell analysis. J. Exp. Med. *205*, 1173–1186.

Laggner, U., Di Meglio, P., Perera, G.K., Hundhausen, C., Lacy, K.E., Ali, N., Smith, C.H., Hayday, A.C., Nickoloff, B.J., and Nestle, F.O. (2011). Identification of a novel proinflammatory human skin-homing Vγ9Vδ2 T cell subset with a potential role in psoriasis. J. Immunol. *187*, 2783–2793.

Lombes, A., Durand, A., Charvet, C., Rivière, M., Bonilla, N., Auffray, C., Lucas, B., and Martin, B. (2015). Adaptive Immune-like γ/δ T lymphocytes share many common features with their α/β T cell counterparts. J. Immunol. *195*, 1449–1458.

Mahtani-Patching, J., Neves, J.F., Pang, D.J., Stoenchev, K.V., Aguirre-Blanco, A.M., Silva-Santos, B., and Pennington, D.J. (2011). PreTCR and TCRγδ signal initiation in thymocyte progenitors does not require domains implicated in receptor oligomerization. Sci. Signal. *4*, ra47.

Malhotra, N., Narayan, K., Cho, O.H., Sylvia, K.E., Yin, C., Melichar, H., Rashighi, M., Lefebvre, V., Harris, J.E., Berg, L.J., and Kang, J.; Immunological Genome Project Consortium (2013). A network of high-mobility group box transcription factors programs innate interleukin-17 production. Immunity *38*, 681–693.

Michel, M.L., Pang, D.J., Haque, S.F., Potocnik, A.J., Pennington, D.J., and Hayday, A.C. (2012). Interleukin 7 (IL-7) selectively promotes mouse and human IL-17-producing γδ cells. Proc. Natl. Acad. Sci. U S A *109*, 17549–17554.

Muñoz-Ruiz, M., Ribot, J.C., Grosso, A.R., Gonçalves-Sousa, N., Pamplona, A., Pennington, D.J., Regueiro, J.R., Fernández-Malavé, E., and Silva-Santos, B. (2016). TCR signal strength controls thymic differentiation of discrete proinflammatory γδ T cell subsets. Nat. Immunol. *17*, 721–727.

Pang, D.J., Neves, J.F., Sumaria, N., and Pennington, D.J. (2012). Understanding the complexity of γδ T-cell subsets in mouse and human. Immunology *136*, 283–290.

Prinz, I., Sansoni, A., Kissenpfennig, A., Ardouin, L., Malissen, M., and Malissen, B. (2006). Visualization of the earliest steps of gammadelta T cell development in the adult thymus. Nat. Immunol. *7*, 995–1003.

Prinz, I., Silva-Santos, B., and Pennington, D.J. (2013). Functional development of γδ T cells. Eur. J. Immunol. *43*, 1988–1994.

Ribot, J.C., deBarros, A., Pang, D.J., Neves, J.F., Peperzak, V., Roberts, S.J., Girardi, M., Borst, J., Hayday, A.C., Pennington, D.J., and Silva-Santos, B. (2009). CD27 is a thymic determinant of the balance between interferon-gamma- and interleukin 17-producing gammadelta T cell subsets. Nat. Immunol. *10*, 427–436.

Schmolka, N., Serre, K., Grosso, A.R., Rei, M., Pennington, D.J., Gomes, A.Q., and Silva-Santos, B. (2013). Epigenetic and transcriptional signatures of stable versus plastic differentiation of proinflammatory γδ T cell subsets. Nat. Immunol. *14*, 1093–1100.

Shibata, K., Yamada, H., Nakamura, M., Hatano, S., Katsuragi, Y., Kominami, R., and Yoshikai, Y. (2014). IFN-γ-producing and IL-17-producing γδ T cells differentiate at distinct developmental stages in murine fetal thymus. J. Immunol. *192*, 2210–2218.

Turchinovich, G., and Hayday, A.C. (2011). Skint-1 identifies a common molecular mechanism for the development of interferon-γ-secreting versus interleukin-17-secreting γδ T cells. Immunity *35*, 59–68.

Wencker, M., Turchinovich, G., Di Marco Barros, R., Deban, L., Jandke, A., Cope, A., and Hayday, A.C. (2014). Innate-like T cells straddle innate and adaptive immunity by altering antigen-receptor responsiveness. Nat. Immunol. *15*, 80–87.

Cell Reports, Volume *19*

Supplemental Information

Strong TCRγδ Signaling Prohibits Thymic Development of IL-17A-Secreting γδ T Cells

Nital Sumaria, Capucine L. Grandjean, Bruno Silva-Santos, and Daniel J. Pennington

Supplementary Figure 1

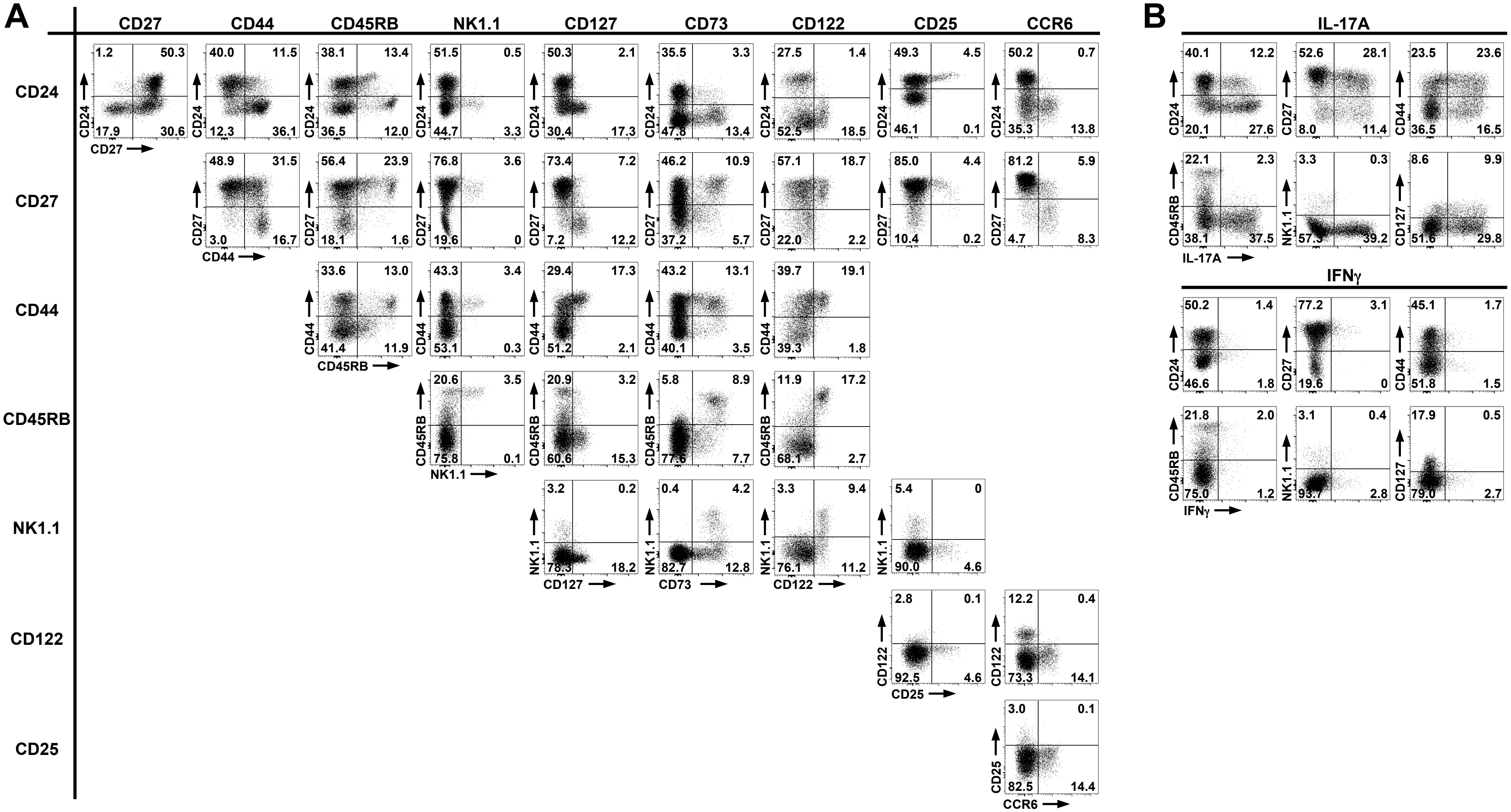

Figure S1. Combinatorial analysis of previously reported surface markers on thymic γδ T cells, Related to Figure 1. (A) Flow cytometry plots of γδ (TCRδ$^{(+)}$CD3ε$^{(+)}$) T cells from the thymus of neonatal C57BL/6 mice (*n* ≥ 5), stained for CD24, CD27, CD44, CD45RB, NK1.1, CD127, CD73, CD122, CD25 and CCR6. **(B)** Plots show thymic γδ T cells stained intracellularly for IL-17A and IFNγ in combination with indicated surface markers. Percentages of cells are indicated in each quadrant.

Supplementary Figure 2

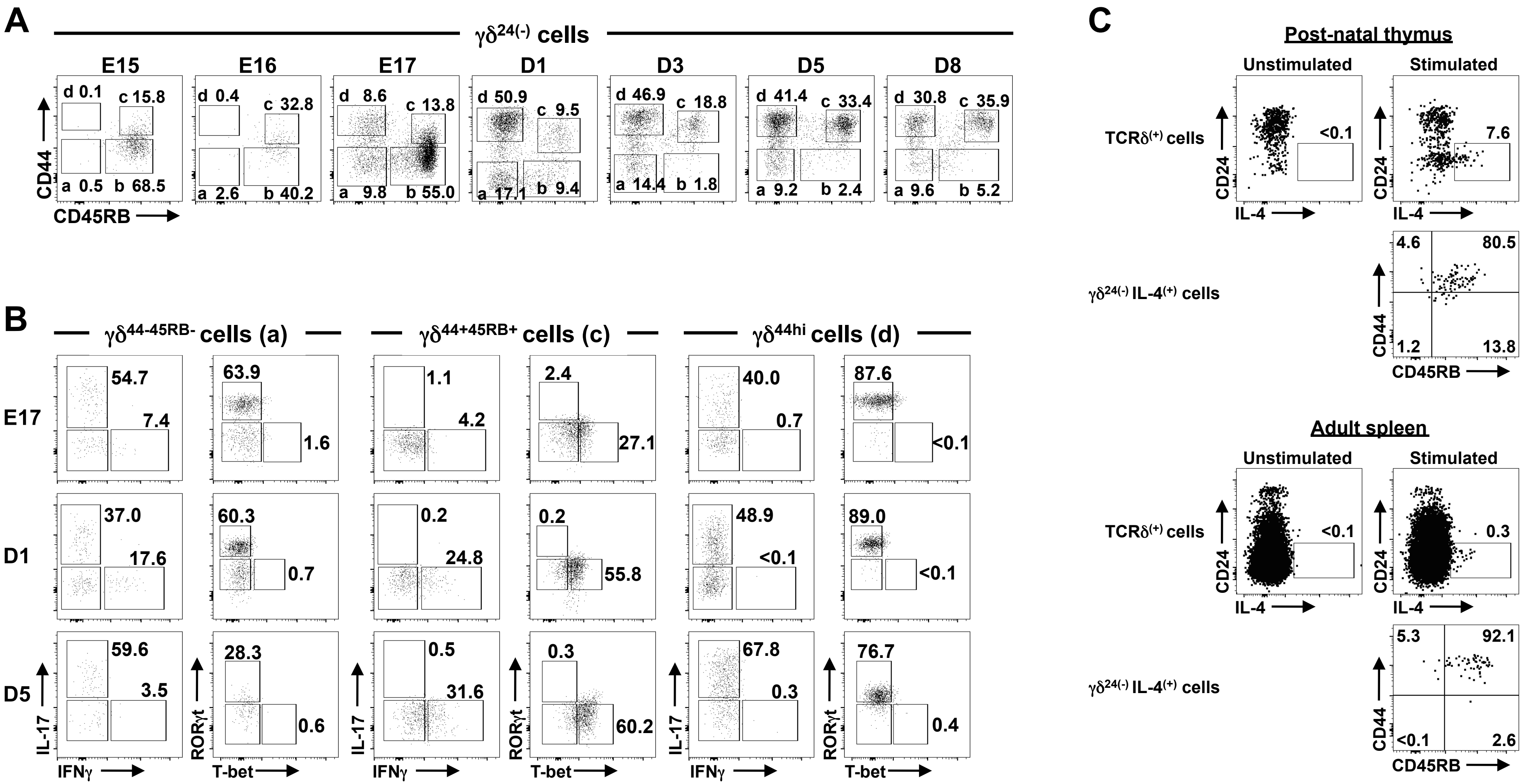

Figure S2. Thymic development of mature γδ T cells and the acquisition of effector function followed through ontogeny, Related to Figure 1. (A) Flow cytometry plots of γδ (TCRδ$^{(+)}$CD3ε$^{(+)}$) T cells in the thymus through ontogeny from E15 to 8-day old B6 mice (*n* ≥ 4 per time-point). CD44/CD45RB plots show CD24$^{(-)}$ γδ T cells. **(B)** Intracellular IL-17A/IFNγ, or RORγt/T-bet, in CD44$^{(-)}$CD45RB$^{(-)}$ (left panel), CD44$^{(+)}$CD45RB$^{(+)}$ (middle panel) and CD44$^{(hi)}$CD45RB$^{(-)}$ (right panel) thymic γδ T cells through ontogeny including E17, 1-day old and 5-day old mice. For cytokines, cells were stimulated 4h *ex vivo* with PMA/ionomycin. **(C)** γδ T cells committed to IL-4 secretion map onto the CD45RB developmental pathway. Total TCRδ$^{(+)}$ thymocytes (top panel) or splenocytes (bottom panel) were sorted from post-natal B6 mice or adult pTα$^{-/-}$ mice, respectively, and stimulated for 18h with PMA/ionomycin or left unstimulated. Top plots in each panel show surface expression of CD24 and intracellular IL-4 staining of γδ T cells. Bottom plots in each panel show surface expression of CD44 and CD45RB on IL-4$^{(+)}$ CD24$^{(-)}$ γδ T cells. Percentages of cells are indicated for each gate or quadrant.

Supplementary Figure 3

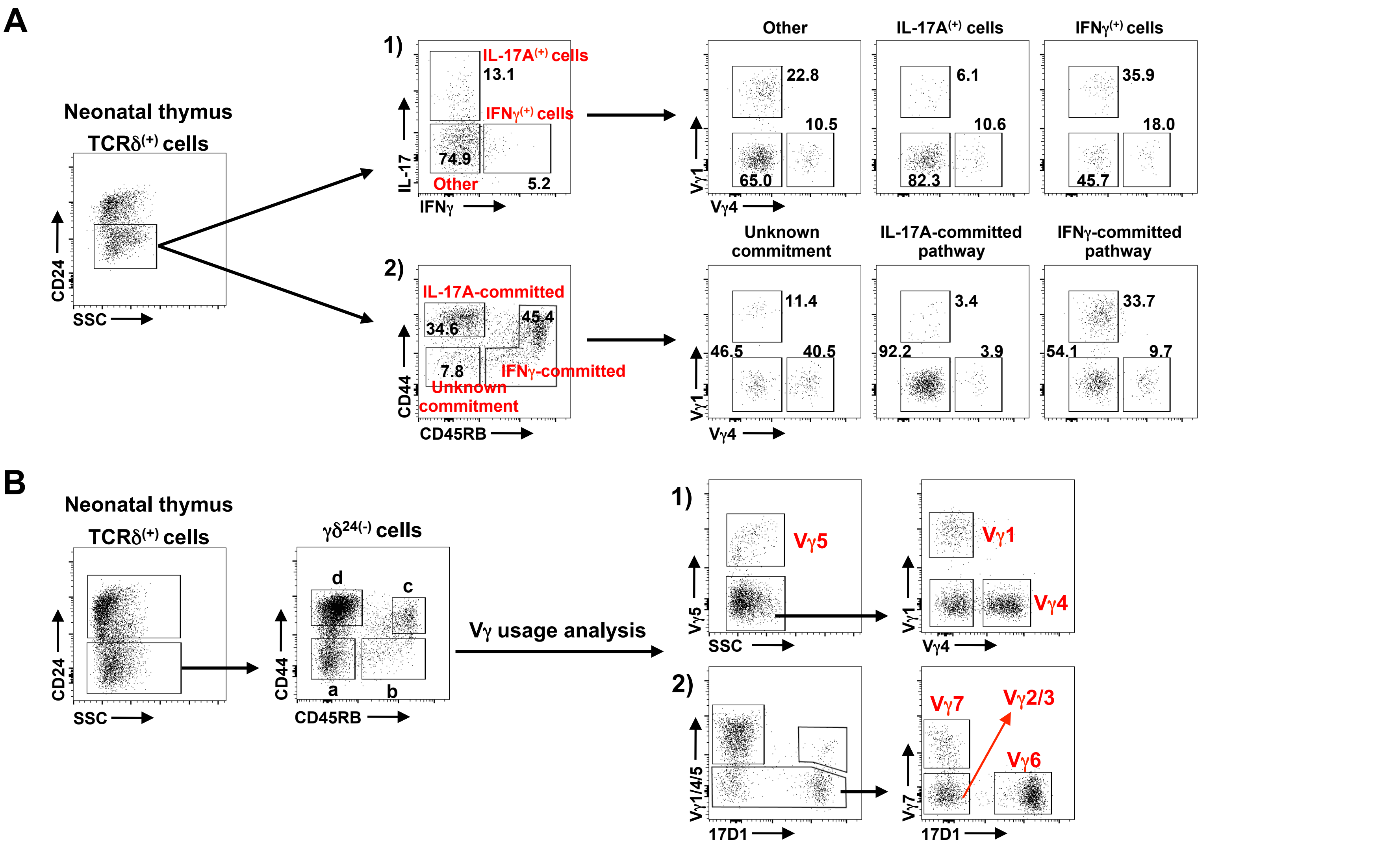

Figure S3. Comparison between gating strategies to identify γδ T cell subsets committed to IL-17A or IFNγ secretion, and specific Vγ usage, Related to Figure 2 and Figure 3. (A) Flow cytometry plots of thymic γδ T cells from neonatal B6 mice stained for CD24, CD44, CD45RB, Vγ1 and Vγ4, followed by intracellular staining for IL-17A and IFNγ. Two gating strategies are depicted: 1) CD24$^{(-)}$ γδ T cells (left) first gated for intracellular IL-17A and IFNγ expression, followed by Vγ-usage (top panels, right) and, 2) CD24$^{(-)}$ γδ T cells (left) first gated for CD44 and CD45RB expression, followed by Vγ-usage (bottom panels, right). Percentages of gated cells are indicated. **(B)** Thymic γδ T cells from neonatal mice were stained using two protocols to identify cells expressing specific Vγ-chains: 1) CD24$^{(-)}$ γδ T cells directly assessed for Vγ1, Vγ4 and Vγ5, and: 2) CD24$^{(-)}$ γδ T cells first gated for Vγ1$^{(-)}$Vγ4$^{(-)}$Vγ5$^{(-)}$ cells followed by analysis for Vγ7 and Vγ6 (using antibody 17D1 that recognises both Vγ5$^{(+)}$ and Vγ6$^{(+)}$ cells).

Supplementary Figure 4

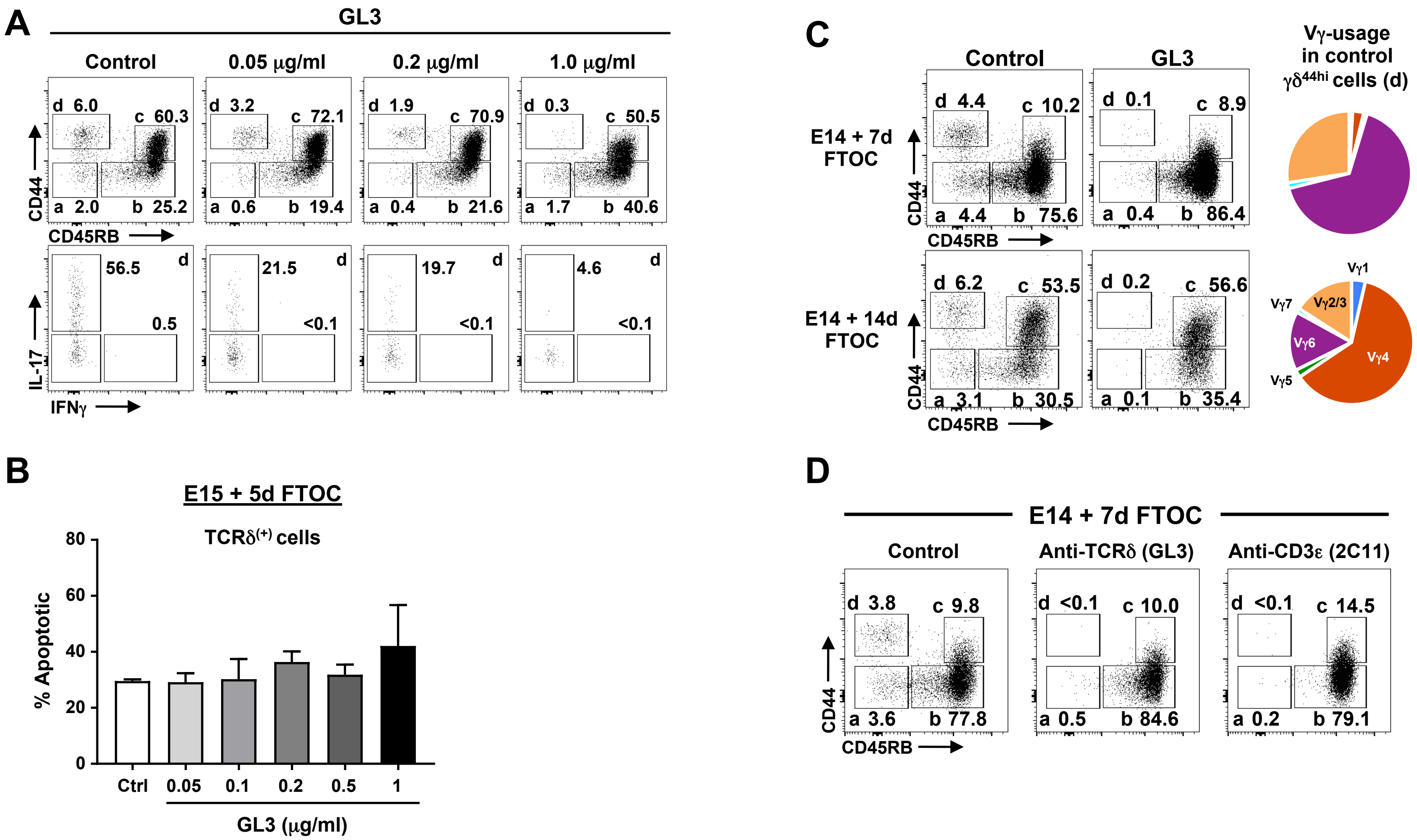

Figure S4. Strong TCRγδ signalling prohibits development of IL-17A-committed γδ T cells, Related to Figure 4. (A) CD44/CD45RB plots (top row) of CD24$^{(-)}$ γδ T cells from 7-day FTOC of E15 B6 thymic lobes in the presence (concentrations as indicated) or absence of anti-TCRδ antibody GL3. Bottom plots show intracellular staining of CD44$^{(hi)}$CD45RB$^{(-)}$ "d" γδ T cells for IL-17A and IFNγ. **(B)** Bar graph shows percentage apoptosis of total γδ T cells from 5-day FTOC of E15 B6 thymic lobes in the presence (concentrations indicated) or absence of GL3. **(C)** CD44/CD45RB (left) on CD24$^{(-)}$ γδ T cells from E14 7-14-day FTOC +/- GL3 (1µg/ml). Vγ-usage by CD44$^{(hi)}$CD45RB$^{(-)}$ γδ T cells is summarised in the pie-charts (right). **(D)** CD44/CD45RB on CD24$^{(-)}$ γδ T cells from 7-day FTOC of E14 B6 thymic lobes in the presence of anti-TCRδ antibody GL3 (1µg/ml), anti-CD3ε antibody 2C11 (1µg/ml), or under control conditions. Percentages of gated cells are indicated. Summarized data are represented as mean ± s.d.

SUPPLEMENTAL EXPERIMENTAL PROCEDURES

Mice: C57BL/6 (B6) mice were purchased from Charles River Laboratories. pTα mutant mice (pTα$^{-/-}$) on a C57BL/6 background were bred in-house. All mice were foetal (E14-E17), neonatal (1-3 days), post-natal (4-8 days) or adult (8-12 weeks). Embryos were obtained by setting up timed pregnancies. Mice were bred and maintained in the specific pathogen-free animal facilities at Queen Mary University of London or relevant facility. All experiments involving animals were performed in full compliance with UK Home Office regulations and institutional guidelines.

Tissue processing and cell isolation: Isolation of lymphocytes from peripheral lymph nodes (axillary, inguinal and brachial) and spleens involved teasing the organs apart, followed by filtering through an 80μm stainless steel mesh (Sefar Ltd., UK) in fluorescence-activated cell sorting (FACS) buffer [phosphate-buffered saline (PBS) containing 2% heat-inactivated foetal calf serum (FCS; Invitrogen) and 5mM ethylenediaminetetraacetic acid (EDTA; Invitrogen)], to obtain single-cell suspensions. Erythrocytes were osmotically lysed in ACK lysis buffer (Invitrogen) and cells washed twice in FACS buffer. Single-cell suspensions of foetal, neonatal and post-natal thymocytes were obtained by gently homogenizing thymic lobes followed by straining through 40μm strainers (BD).

Foetal Thymic Organ Cultures (FTOC): E14 or E15 thymic lobes from B6 mice were cultured on nucleopore membrane filter discs (Whatman) in FTOC medium (RPMI-1640 with 10% FCS, 1% penicillin and streptomycin, 50μM β-mercaptoethanol, and 2mM L-glutamine) for 7-14 days (unless otherwise indicated). In some experiments anti-TCRδ antibody (GL3; 1μg/ml unless otherwise indicated), anti-CD3ε antibody (2C11; 1 μg/ml unless otherwise indicated), or MEK1/2 inhibitor UO126 (5μM; Sigma-Aldrich) were added to the cultures. Cultures containing antibody or inhibitor were rested overnight in fresh FTOC medium before analysis. All thymic organ cultures were subsequently analysed by flow cytometry.

In vivo treatment with monoclonal anti-CD3ε antibody: Time-mated pregnant WT mice at 13-days post-conception were injected intraperitoneally (i.p.) with anti-CD3ε antibody (2C11; 40 μg in 500 μl PBS per mouse) or PBS only, and allowed to give birth. Thymocytes were isolated from 2-day old neonatal mice as described above, and processed for analysis by flow cytometry.

Cell sorting and OP9-DL1 co-cultures: The OP9-DL1 cell line was provided by J.C Zúniga-Pflücker (University of Toronto, Canada) (Schmitt and Zuniga-Pflucker, 2002). OP9-DL1 cells were maintained in Dulbecco's modified Eagles's medium (DMEM) with GlutaMAX (Invitrogen), 10% FCS, 1% penicillin and streptomycin, 50 μM β-mercaptoethanol (Invitrogen), and 1% non-essential amino acids (Invitrogen) at 37°C and 5% CO_2. OP9-DL1 cells were passaged 1:4 or 1:5 every 2 days and were maintained at 75% confluency. Thymocytes from B6 E15 thymic lobes cultured for 7 days were isolated (as described above), stained and sorted under sterile conditions on a FACSAria (BD) into the four mature γδ$^{24(-)}$ subsets i.e. CD44$^{(-)}$CD45RB$^{(-)}$ "a", CD44$^{(-)}$CD45RB$^{(+)}$ "b", CD44$^{(+)}$CD45RB$^{(+)}$ "c", and CD44$^{(hi)}$CD45RB$^{(-)}$ "d". Sorted cells were seeded in triplicate onto a semi-confluent monolayer of OP9-DL1 cells cultured in flat-bottom 96-well plates with 200 μl of OP9-DL1 medium supplemented with 5 ng/ml Flt3 ligand (Miltenyi Biotec) and 1 ng/ml interleukin-7 (IL-7; Miltenyi Biotec). IL-7 and Flt3 ligand were replenished every 3 days. Cells were incubated for 5 days at 37°C and 5% CO_2. Cultured cells were subsequently analysed by flow cytometry.

Flow cytometry: Fluorochrome-conjugated antibodies (purchased from eBioscience, BD or Biolegend) against the following cell surface molecules and cytokines were used: CCR6 (29-2L17), CD3ε (145-2C11), CD24 (M1/69), CD25 (PC61), CD27 (LG.7F9), CD44 (IM7), CD45RB (C363.16A), CD73 (TY/11.8), CD122 (TM-b1), CD127 (IL-7R; SB/199), NK1.1 (PK136), TCRδ (GL3), Vγ1 (2.11), Vγ4 (UC3-10A6), Vγ5 (536), Vγ7 (F2.67; kindly provided by Dr Pablo Pereira), IFNγ (XMG1.2), IL-4 (11B11) and IL-17A (eBio17B7). 17D1 antibody (rat IgM) that recognises Vγ5Vδ1 and Vγ6Vδ1 was kindly provided by Prof Adrian Hayday as hybridoma supernatant which is used as the primary staining reagent, followed by staining with fluorochrome-conjugated anti-rat IgM antibody. For cell surface staining, thymocytes and lymphocytes were incubated on ice with anti-CD16/CD32 (2.4G2; eBioscience) to block Fc receptors and stained with fluorochrome-conjugated antibodies diluted in FACS buffer. For detection of both Vγ5Vδ1 and Vγ6Vδ1, cells were pre-stained with anti-TCRδ (GL3) antibody at room temperature followed by staining with 17D1. After staining, cells were washed and re-suspended in FACS buffer containing 0.5μg/ml DAPI (Invitrogen) for dead cell exclusion prior to analysis. For intracellular cytokine staining, cells were

stimulated with 50ng/ml phorbol 12-myristate 13-acetate (PMA; Sigma) and 1μg/ml ionomycin (Sigma) for 4h (or 18h for IL-4 detection) at 37°C; 10μg/ml Brefeldin A (eBioscience) and 2μM Monensin (eBioscience) were added during the last 2h (or 4h for IL-4 detection). Cells were stained for cell surface markers and Zombie Aqua™ Fixable Viability dye (Biolegend) for dead cell exclusion, fixed with IC fixation buffer (eBioscience) for 15min on ice and subsequently permeabilised and stained with intracellular cytokine-specific antibodies diluted in permeabilisation buffer (eBioscience). For intracellular detection of transcription factors, cells were fixed and permeabilised with the Foxp3/Transcription Factor buffer set (eBioscience) as per manufacturer's instructions and subsequently stained with antibody to RORγt (B2D; eBioscience) and T-bet (4B10; eBioscience). For detection of apoptosis, cells were stained with Annexin V (Biolegend) followed by the addition of DAPI (0.05 μg/ml) prior to sample acquisition. Samples were acquired using an LSR-II flow cytometer (BD) or Canto II (BD) and data were analysed using FlowJo software (Tree Star, Inc.).

Statistical analysis: Data are presented as mean ± s.d. Student's *t*-test or one-way analysis of variance (ANOVA) were used to assess statistical significance of differences between groups. A difference was considered significant if $P \leq 0.05$.

References:

Schmitt, T.M., and Zuniga-Pflucker, J.C. (2002). Induction of T cell development from hematopoietic progenitor cells by delta-like-1 *in vitro*. Immunity *17*, 749-756.
